# Author Correction: Morphogenetic metasurfaces: unlocking the potential of Turing patterns

**DOI:** 10.1038/s41467-024-47832-1

**Published:** 2024-05-02

**Authors:** Thomas Fromenteze, Okan Yurduseven, Chidinma Uche, Eric Arnaud, David R. Smith, Cyril Decroze

**Affiliations:** 1 University of Limoges, CNRS, XLIM, UMR 7252, F-87000 Limoges, France; 2 Centre for Wireless Innovation (CWI), Institute of Electronics, Communications and Information Technology (ECIT), Queen’s University Belfast, Belfast, BT3 9DT UK; 3 Center for Metamaterials and Integrated Plasmonics, Department of Electrical and Computer Engineering, Duke University, Durham, NC 27708 USA

**Keywords:** Electrical and electronic engineering, Metamaterials

Correction to: *Nature Communications* 10.1038/s41467-023-41775-9, published online 06 October 2023

1. Error location: Missing symbols - within a single Eq. (3).

Error: missing symbol in the second component of Eq. (3) of the Article (identically, Eq. (10) from Supplementary Information).

Correction text: The original version of this Article contained an error in Eq. (3) (Eq. (10) from Supplementary Information). The second component of the reactance tensor must include the average reactance *X*_*sw*_, and incorrectly read:

$${{{{\bf{X}}}}}_{\rho \rho }=	{X}_{sw}(1+{a}_{X}^{\max }{{{{\bf{M}}}}}_{\rho \rho }\,\sin ({{\mbox{arg}}}({{{{\bf{E}}}}}_{\rho }/{{{{\bf{J}}}}}_{\rho })))\\ {{{{\bf{X}}}}}_{\rho \phi }=	{a}_{X}^{\max }{{{{\bf{M}}}}}_{\rho \phi }\,\sin ({{\mbox{arg}}}({{{{\bf{E}}}}}_{\phi }/{{{{\bf{J}}}}}_{\rho }))\\ {{{{\bf{X}}}}}_{\phi \rho }=	{{{{\bf{X}}}}}_{\rho \phi }\\ {{{{\bf{X}}}}}_{\phi \phi }=	{X}_{sw}(1-{a}_{X}^{\max }{{{{\bf{M}}}}}_{\rho \rho }\,\sin ({{\mbox{arg}}}({{{{\bf{E}}}}}_{\rho }/{{{{\bf{J}}}}}_{\rho }))).$$

The correct form of Eq. (3) is:

$${{{{\bf{X}}}}}_{\rho \rho }=	{X}_{sw}(1+{a}_{X}^{\max }{{{{\bf{M}}}}}_{\rho \rho }\,\sin ({{\mbox{arg}}}({{{{\bf{E}}}}}_{\rho }/{{{{\bf{J}}}}}_{\rho })))\\ {{{{\bf{X}}}}}_{\rho \phi }=	{X}_{sw}{a}_{X}^{\max }{{{{\bf{M}}}}}_{\rho \phi }\,\sin ({{\mbox{arg}}}({{{{\bf{E}}}}}_{\phi }/{{{{\bf{J}}}}}_{\rho }))\\ {{{{\bf{X}}}}}_{\phi \rho }=	{{{{\bf{X}}}}}_{\rho \phi }\\ {{{{\bf{X}}}}}_{\phi \phi }=	{X}_{sw}(1-{a}_{X}^{\max }{{{{\bf{M}}}}}_{\rho \rho }\,\sin ({{\mbox{arg}}}({{{{\bf{E}}}}}_{\rho }/{{{{\bf{J}}}}}_{\rho }))).$$

This typo has no impact on the results presented, which are derived from numerical models based on the correct expression of reactance tensors.

### Supplementary information

Updated Supplementary Information

