# Supplementary material for: Morphogenetic metasurfaces: unlocking the potential of Turing patterns
Source: Nat Commun. 2023 Oct 6;14:6249. doi: 10.1038/s41467-023-41775-9 (PMC10558543; doi:10.1038/s41467-023-41775-9)
Supplement: Supplementary file 1 — Supplementary Information [file 41467_2023_41775_MOESM1_ESM.pdf]

Morphogenetic Metasurfaces: Unlocking the Potential of Turing Patterns
Supplementary information

Thomas Fromenteze,[1,*] Okan Yurduseven,[2] Chidinma Uche,[1] Eric Arnaud,[1] David R. Smith,[3] and Cyril Decroze[1]

[1,*] University of Limoges, CNRS, XLIM, UMR 7252, F-87000 Limoges, France

[2] Centre for Wireless Innovation (CWI), Institute of Electronics, Communications and Information Technology (ECIT), Queen's University Belfast, Belfast, BT3 9DT, UK

[3] Center for Metamaterials and Integrated Plasmonics, Department of Electrical and Computer Engineering, Duke University, Durham, NC 27708, USA

[*] Corresponding author: thomas.fromenteze@unilim.fr

1 Definition of objective reactance tensors

The generation of metasurfaces must be constrained by the definition of objective electromagnetic parameters. A vertical monopole placed in the center of this structure radiates a dominantly transverse magnetic wave, guided between two copper plates within a dielectric substrate. The etching of patterns on the upper surface enables the conversion of surface waves into radiated waves by means of reactance tensors that must be modulated over the entire metasurface. The definition of objective tensors requires to constrain both the incident waves and the radiated ones, exploiting the following relation [1]:

$$\mathbf{E}_t|_{z=0^+} = \underline{\underline{\mathbf{Z}}}(\omega, \mathbf{k}_t) \cdot \big([\hat{\mathbf{z}} \times \mathbf{H}_t]_{z=0^+} - [\hat{\mathbf{z}} \times \mathbf{H}_t]_{z=0^-}\big). \tag{1}$$

A series of approximations is considered in order to facilitate this demonstration as much as possible. The flexibility of the proposed synthesis method allows nevertheless to reach realized gains close to the state of the art, obtained following more advanced mathematical models and restricted metasurface shapes. On the one hand, the ohmic losses of the etched patterns are not considered, obtaining impedance tensors with a zero real part and reducing to the reactance terms only:

$$\underline{\underline{\mathbf{Z}}} = j\underline{\underline{\mathbf{X}}}. \tag{2}$$

In practice, the synthesized electromagnetic properties are obtained far enough from resonance phenomena to minimize the impact of resistive losses. We then consider that the electromagnetic properties of a metasurface do not depend on the angle of incidence of the plane waves emitted or received by the latter, implying:

$$\underline{\underline{\mathbf{X}}}(\omega, \mathbf{k}_t) = \underline{\underline{\mathbf{X}}}(\omega). \tag{3}$$

The characterizations will thus be carried out by excitation of plane waves at normal incidence, potentially limiting the performance of the metasurfaces for strong beam steering where the reactance values can be altered. As a last approximation, we consider that the incident waves excited by the monopole at the center of the metasurface are weakly perturbed by the reactance modulation. We also consider that the conversion to radiated waves is sufficiently efficient to neglect the reflected contributions at the metasurface boundaries. Under these conditions, the incident magnetic wave generated by the monopole no longer depends on the geometry of the metasurface, providing a first-order interaction model:

$$\mathbf{E}_t|_{z=0^+} = j\underline{\underline{\mathbf{X}}} \cdot \mathbf{J}, \tag{4}$$

with

$$\mathbf{J} = J_0\, H_1^{(2)}(-jk_{sw}\boldsymbol{\rho})\hat{\boldsymbol{\rho}} = \mathbf{J}_\rho\, \hat{\boldsymbol{\rho}}. \tag{5}$$

The wave vector $k_{sw} = \alpha_{sw} + j\beta_{sw}$ reflects by its real part the losses by radiation (and beyond our approximation, by ohmic effect) and its imaginary part defines the average phase delay cumulated per unit length by the incident wave. This last quantity depends directly on the average surface reactance X_{sw} exhibited by the printed patterns:

$$\beta_{sw} = k_0\sqrt{1 + \left(\frac{X_{sw}}{\eta_0}\right)^2}, \tag{6}$$

where k_0 and η_0 represent respectively the wavenumber and the free space impedance. For this work, the patterns will be generated to maintain a constant average reactance X_{sw}, thus determining the spatial frequencies of the printed patterns and guaranteeing the adaptation of the monopole exciting the reference wave.

The design of metasurfaces then requires the determination of radiation objectives. Some specifications, in particular defined in far field and for a restricted combination of plane waves, facilitate the definition of analytical formalisms of objective reactance tensors. To propose a generative model as flexible as possible, an arbitrary radiation objective $\mathbf{E}(\mathbf{r})$ is considered for the rest of these explanations. This field is radiated by

fictitious magnetic currents $\mathbf{M}_s(\mathbf{r}')$ through the free space dyadic Green's function $\underline{\underline{\mathbf{G}}}(\mathbf{r}, \mathbf{r}')$ such that:

$$\mathbf{E}(\mathbf{r}) = \iint_S [\nabla \times \underline{\underline{\mathbf{G}}}(\mathbf{r}, \mathbf{r}') \cdot \mathbf{M}_s(\mathbf{r}')] dS'. \tag{7}$$

These magnetic sources are determined from the tangential field of the metasurface:

$$\mathbf{M}_s = -\hat{\mathbf{z}} \times \mathbf{E}_t|_{z=0^+}. \tag{8}$$

The definition of a radiation objective is finally achieved by spatially discretizing the radiating surface and the region of interest to define a propagation operator $\underline{\underline{\mathbf{G}}}_{ee}$ such that:

$$\mathbf{E} = \underline{\underline{\mathbf{G}}}_{ee} \cdot \mathbf{E}_t. \tag{9}$$

Following the definition of this forward problem, the tangential electric field on the metasurface allowing the satisfaction of this radiation objective is finally determined by pseudo-inversion of $\underline{\underline{\mathbf{G}}}_{ee}$, corresponding to a back-propagation of the objective field towards the radiating aperture. To simplify again this demonstration, the only control of tangential fields is studied but more complete methods can be considered for the synthesis of radiation objectives by including longitudinal elements corresponding to the control of currents of electric nature [2]. These contributions have not been retained in this model because they weakly affect the radiation of the metasurface outside of large incidence angles.

According to one of the founding papers of radiating metasurfaces [3], a holographic approach is considered in order to facilitate the definition of reactance tensors. Following Eq. (4), the objective is thus to synthesize a surface impedance able to transform a reference wave, corresponding here to the currents $\mathbf{J}$, into a radiated wave, defined just above the surface by $\mathbf{E}_t|_{z=0^+}$. The reactance tensor to be synthesized being by definition of higher rank than the transformed vectors, there is no unique definition of its components and additional constraints can be considered to facilitate its derivation. First of all, the reactance tensors $\underline{\underline{\mathbf{X}}}$ satisfy hermiticity conditions by forcing them to contain real and symmetric coefficients ($\underline{\underline{\mathbf{X}}} = \underline{\underline{\mathbf{X}}}^T$). In this way, they are decomposable into orthonormal eigenvectors, associated to real eigenvalues defining the anisotropy of each reactance tensor. It is also necessary to control the mean value of the reactance tensors, associated to the sum of its diagonal elements and corresponding to the trace operator $\mathrm{Tr}(\underline{\underline{X}}) = X_{sw}$. Following a holographic approach, a phase matching is finally realized by a modulation allowing the compensation of the phase of the reference wave and the creation of the

objective one:

$$\begin{aligned}
\mathbf{X}_{\rho\rho} &= X_{sw}\big(1 + a_X^{\max}\,\mathbf{M}_{\rho\rho}\,\sin(\arg(\mathbf{E}_\rho/\mathbf{J}_\rho))\big)\\
\mathbf{X}_{\rho\phi} &= X_{sw}\,a_X^{\max}\,\mathbf{M}_{\rho\phi}\,\sin(\arg(\mathbf{E}_\phi/\mathbf{J}_\rho))\\
\mathbf{X}_{\phi\rho} &= \mathbf{X}_{\rho\phi}\\
\mathbf{X}_{\phi\phi} &= X_{sw}\big(1 - a_X^{\max}\,\mathbf{M}_{\rho\rho}\,\sin(\arg(\mathbf{E}_\rho/\mathbf{J}_\rho))\big).
\end{aligned} \tag{10}$$

The amplitude modulation of the generated hologram plays an important role in the conversion efficiency of the reference wave into a radiated wave [4, 5]. The objective is thus to couple independently the orthogonal components of an incident wave along the preferred directions corresponding to the eigenvectors of the reactance tensor at each point of the metasurface (Fig. S1).

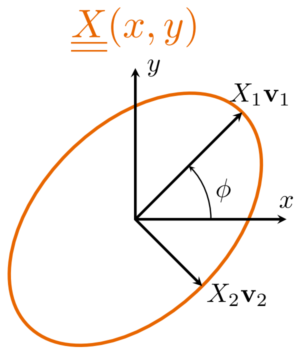

Figure S1: Anisotropy of a reactance tensor represented by an ellipse whose principal axes correspond to the eigenvectors of the tensor weighted by the associated eigenvalues. A perfectly isotropic reactance corresponds to identical eigenvalues, thus forming a circle.

At each point of the metasurface, the considered Hermitian formalism allows the following eigenvalue decomposition:

$$\underline{\underline{\mathbf{X}}} = \underline{\underline{\mathbf{R}}}_\phi\,\mathrm{diag}(\mathbf{X}_1, \mathbf{X}_2)\,\underline{\underline{\mathbf{R}}}_\phi^T, \tag{11}$$

where the eigenvalues $\mathbf{X}_1$ and $\mathbf{X}_2$ are defined as follows:

$$\begin{aligned}
\mathbf{X}_1 &= X_{sw}(1 + a_X^{\max}\,\mathbf{M}_{\rho\rho})\\
\mathbf{X}_2 &= X_{sw}(1 - a_X^{\max}\,\mathbf{M}_{\rho\rho}).
\end{aligned} \tag{12}$$

The tensors $\underline{\underline{\mathbf{R}}}_\phi$ correspond to rotation matrices of an angle ϕ with respect to the reference coordinate system, as represented in Fig. S1. A direct analogy exists with the Jones matrices defined for metasurfaces used in transmission, allowing advanced control

of polarization conversion [6].

The anisotropy of the reactance tensors is finally defined by the constant $a_X^{\max}$, determined as a function of the values achievable by the synthesized patterns, as developed in the next section. A modulation of the anisotropy is performed in order to satisfy the polarization constraints of the reference and objective fields. The distributions $\mathbf{M}_{\rho\rho} \in [0, 1]$ and $\mathbf{M}_{\rho\phi} \in [0, 1]$ are thus determined by normalizing the magnitude of the following relations:

$$\mathbf{M}_{\rho\rho} = \left(\left| \frac{\mathbf{E}_\rho}{\mathbf{J}_\rho} \right| / \max \left(\left| \frac{\mathbf{E}}{\mathbf{J}} \right| \right) \right)^{\alpha} \tag{13}$$

$$\mathbf{M}_{\rho\phi} = \left(\left| \frac{\mathbf{E}_\phi}{\mathbf{J}_\rho} \right| / \max \left(\left| \frac{\mathbf{E}}{\mathbf{J}} \right| \right) \right)^{\alpha} \tag{14}$$

The α parameter (set at $\alpha = 0.5$ in this work) finally allows the adjustment of the spatial variation of anisotropy. By matching the decay of the surface wave with an increasing reactance modulation, the effective area occupied by the leaky wave can be optimized to increase the gain of the synthesized antennas [4, 5]. It should be noted that the formalism considered for the reference wave, corresponding here to the surface currents induced by a monopole disposed at the center of the metasurface, contains a singularity at the location of the connector. For this demonstration, only the contributions outside the surface occupied by the monopole are retained for the synthesis of objective tensors.

2 Anisotropic pattern synthesis

Following the definition of objective reactance tensors allowing the conversion of a reference wave into one or various radiation objectives, it is now necessary to propose a technique enabling the automated synthesis of patterns able to satisfy the desired electromagnetic constraints. A procedural generation technique is developed in this work leveraging the reaction-diffusion principle introduced by Alan Turing [7].

The principle is based on the interaction between antagonistic chemical species that he referred to as "morphogens", able to diffuse in space and interact to form biological patterns. Following such mechanisms, the Gray-Scott model [8] is harnessed here for its ease of implementation. This dynamic system is associated with the evolution of two populations of morphogens U and V whose reaction is described by :

$$U + 2V \rightarrow 3V \tag{15}$$

$$V \rightarrow P. \tag{16}$$

At each time step, U morphogens are thus converted into V particles. To reach an equilibrium, the V morphogens are also progressively converted into P inert particles. The U and V morphogens also have the ability to diffuse in space over time. These reaction and diffusion mechanisms can be converted into the following differential system:

$$\frac{\partial \mathbf{U}}{\partial t} = \mathbf{d_u}\nabla^2\mathbf{U} - \mathbf{U}\mathbf{V}^2 + \mathbf{f}(1 - \mathbf{U}) \tag{17}$$

$$\frac{\partial \mathbf{V}}{\partial t} = \mathbf{d_v}\nabla^2\mathbf{V} + \mathbf{U}\mathbf{V}^2 - (\mathbf{f} + \mathbf{k})\mathbf{V}. \tag{18}$$

The values $\mathbf{d_u}$ and $\mathbf{d_v}$ correspond to the diffusion coefficients associated with the Laplacian operator ∇^2. $\mathbf{f}$ (feed) represent the spontaneous generation rate of morphogens U and $\mathbf{k}$ (kill) corresponds to an extinction rate of the morphogens V.

The common term $\mathbf{UV}^2$ corresponds to the probability of conversion of a morphogen U by two V, thus subtracted from the concentrations $\mathbf{U}$ and added to the concentrations $\mathbf{V}$.

Depending on the value of $\mathbf{f}$, the continuous emergence of morphogen U is controlled by applying the generation rate to the term $(1 - \mathbf{U})$. Following a proper initialization, it is thus not possible to exceed a concentration of 1 because this term becomes less and less active as $\mathbf{U}$ increases.

For the same purpose of morphogen population control, it is necessary that V elements are removed faster than U elements are created, making the total exctinction rate always

greater than the generation rate via the term $-(\mathbf{f}+\mathbf{k})\mathbf{V}$.

Although a Lagrangian description of the behavior of individual particles facilitates the understanding of the interactions between morphogens, it is preferable to consider an Eulerian approach for solving the associated differential system. We reduce the problem to a set of spatially discretized concentrations $\mathbf{U}$ and $\mathbf{V}$ denoted in bold and defined on a uniform grid forming the pixels of the generated patterns. An explicit Euler scheme is implemented for the resolution of this system, allowing at each iteration to ensure the growth of the Turing patterns:

$$\mathbf{U_{t+1}} = \mathbf{U_t} + \Delta t\left(\mathbf{d_u}\nabla^2\mathbf{U_t} - \mathbf{U_t}\mathbf{V_t}^2 + \mathbf{f}(1-\mathbf{U_t})\right) \tag{19}$$

$$\mathbf{V_{t+1}} = \mathbf{V_t} + \Delta t\left(\mathbf{d_v}\nabla^2\mathbf{V_t} + \mathbf{U_t}\mathbf{V_t}^2 - (\mathbf{f}+\mathbf{k})\mathbf{V_t}\right). \tag{20}$$

The resolution is therefore based on a discretization of time and space and facilitates the implementation of the diffusion operator. The Laplacian operator can indeed be approximated in a finite difference scheme by the five-point stencil method:

$$\nabla^2\mathbf{U_t}|_{x_i,y_j} = \frac{\partial^2\mathbf{U_t}}{\partial x^2}|_{x_i,y_j} + \frac{\partial^2\mathbf{U_t}}{\partial y^2}|_{x_i,y_j} \tag{21}$$

$$\approx \frac{U_{i-1,j} - 2U_{i,j} + U_{i+1,j}}{h^2} + \frac{U_{i,j-1} - 2U_{i,j} + U_{i,j+1}}{h^2} \tag{22}$$

where h corresponds to the spatial step normalized to 1 in our work. The diffusion can thus be computed by linear combination of neighboring elements and potentially accelerated by implementing this computation by convolution of a mask $\underline{\underline{\mathbf{L}}}$ representing the weights applied on these elements, corresponding to a discrete Laplacian operator:

$$\underline{\underline{\mathbf{L}}} = \begin{bmatrix} 0 & 1 & 0 \\ 1 & -4 & 1 \\ 0 & 1 & 0 \end{bmatrix}. \tag{23}$$

This finite difference scheme is the simplest to implement but can be limited by stability issues and anisotropic behavior, favoring the generation of patterns along the vertical and horizontal axes. If the stability can be improved by decreasing the value of the time step Δt at the cost of slower generations, it is still preferable to consider alternatives to the expression of the discrete Laplacian operator including higher-order terms such as the nine-point stencil method:

$$\underline{\underline{\mathbf{L}}} = \begin{bmatrix} 0.05 & 0.2 & 0.05 \\ 0.2 & -1 & 0.2 \\ 0.05 & 0.2 & 0.05 \end{bmatrix}. \tag{24}$$

In order to represent a fraction of the Turing patterns that can be generated by the Gray-Scott model, a finite difference implementation is thus realized on a two dimensional Cartesian domain (Fig. S2).

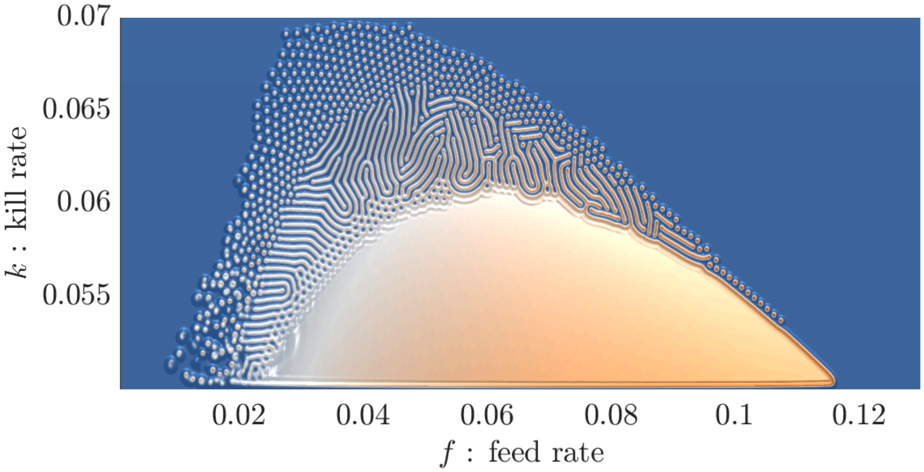

Figure S2: Growth of Turing patterns for different couples of parameters **f** and **k**. The generation is computed with $d_u = 1$, $d_v = 0.5$ and $\Delta t = 1$. The initialization is done by randomly imposing 500 pixels of the matrix **V** at a value of 0.5. The rest of the elements of **V** are initialized to 0 and the whole matrix **U** to a value of 1.

Depending on the values of generation rate f and extinction rate k, some areas are entirely populated by U or V morphogens. At the interface, an equilibrium between the populations for certain couples (f, k) ensures the generation of different patterns. The central role of reaction-diffusion mechanisms is now observed in many contexts related to the structuring of the living. In particular, it is possible to observe a wide variety of Turing patterns on the skin of certain type of fish for purposes that appear to be related to social issues, camouflage and protection from predators. The catfishes of the genus Pseudoplatystoma exhibit among the most important diversity of observable patterns, varying according to the geographical origin of the observed species [9] (Fig. S3).

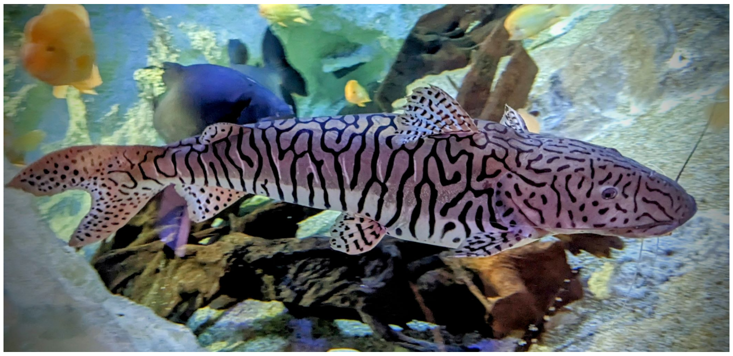

Figure S3: Representation of a variety of Turing patterns on a pseudoplatystoma fasciatum, courtesy of the Aquarium of Limoges, France.

Inspired by the ability of living organisms to locally self-organize in response to external stimuli or to fulfill biological functions, we are interested in exploiting these generative models to ensure the synthesis of structures with desired electromagnetic properties. Many combinations guarantee the generation of circular patterns whose reproduction resembles that of cells, occupying the available space following a succession of divisions. This type of Turing pattern is retained in this work, exploiting its ability to automatically form self-similar and compact arrangements that can be used to ensure local control of reactance on metasurfaces. Overall, this generation technique is also particularly well suited to meet the definition of boundary conditions prohibiting pattern growth. In the example given in Fig. S4, cellular patterns are reproduced by following geometrical constraints on the only authorized domains, finding a compact arrangement as iterations are performed.

In this example, the growth of a Turing pattern is realized by defining boundary conditions allowing to reveal the words ”Gray-Scott”, in reference to the exploited generative model [8]. The synthesis is computed on a domain of 800×200 pixels for a succession of 10 000 iterations in 13s, using a Matlab program executed on a computer equipped with an i9-10900K processor running at 3.70 GHz. The generated pattern quickly converges to a compact arrangement following a set of divisions of the cellular elements whose population grows exponentially during the iterations to occupy the free space.

This generative model thus provides an easy way to impose the types and dimensions of Turing patterns while respecting arbitrary boundary conditions. In connection with the isotropic diffusion of the morphogens computed with the discrete Laplacian operator, the circular shapes of the selected patterns do not however offer independent control of incident orthogonal polarizations by anisotropy effect, being unable to adjust the orientation of the generated elements. In order to break these local symmetries and to impose privileged polarizations for the coupling of waves, it is thus necessary to deform these patterns to make them elliptical. The Gray-Scott model can thus be modified, generating patterns with the help of anisotropic diffusion [10, 11, 12]:

$$\frac{\partial \mathbf{U}}{\partial t} = \mathbf{d_u} \nabla \cdot (\underline{\underline{\mathbf{D}}} \nabla \mathbf{U}) - \mathbf{U}\mathbf{V}^2 + \mathbf{f}(1 - \mathbf{U}) \tag{25}$$

$$\frac{\partial \mathbf{V}}{\partial t} = \mathbf{d_v} \nabla \cdot (\underline{\underline{\mathbf{D}}} \nabla \mathbf{V}) + \mathbf{U}\mathbf{V}^2 - (\mathbf{f} + \mathbf{k})\mathbf{V}. \tag{26}$$

The diffusion is thus carried out by defining for each point of the space an associated tensor $\underline{\underline{\mathbf{D}}}$, decomposing the Laplacian operator into two successive gradients:

$$\nabla \cdot (\underline{\underline{\mathbf{D}}} \nabla \mathbf{M}) = \begin{bmatrix} \frac{\partial}{\partial x} & \frac{\partial}{\partial y} \end{bmatrix} \begin{bmatrix} D_{xx} & D_{xy} \\ D_{yx} & D_{yy} \end{bmatrix} \begin{bmatrix} \frac{\partial \mathbf{M}}{\partial x} \\ \frac{\partial \mathbf{M}}{\partial y} \end{bmatrix}.$$

Considering an illustration similar to that proposed for the analysis of the reactance

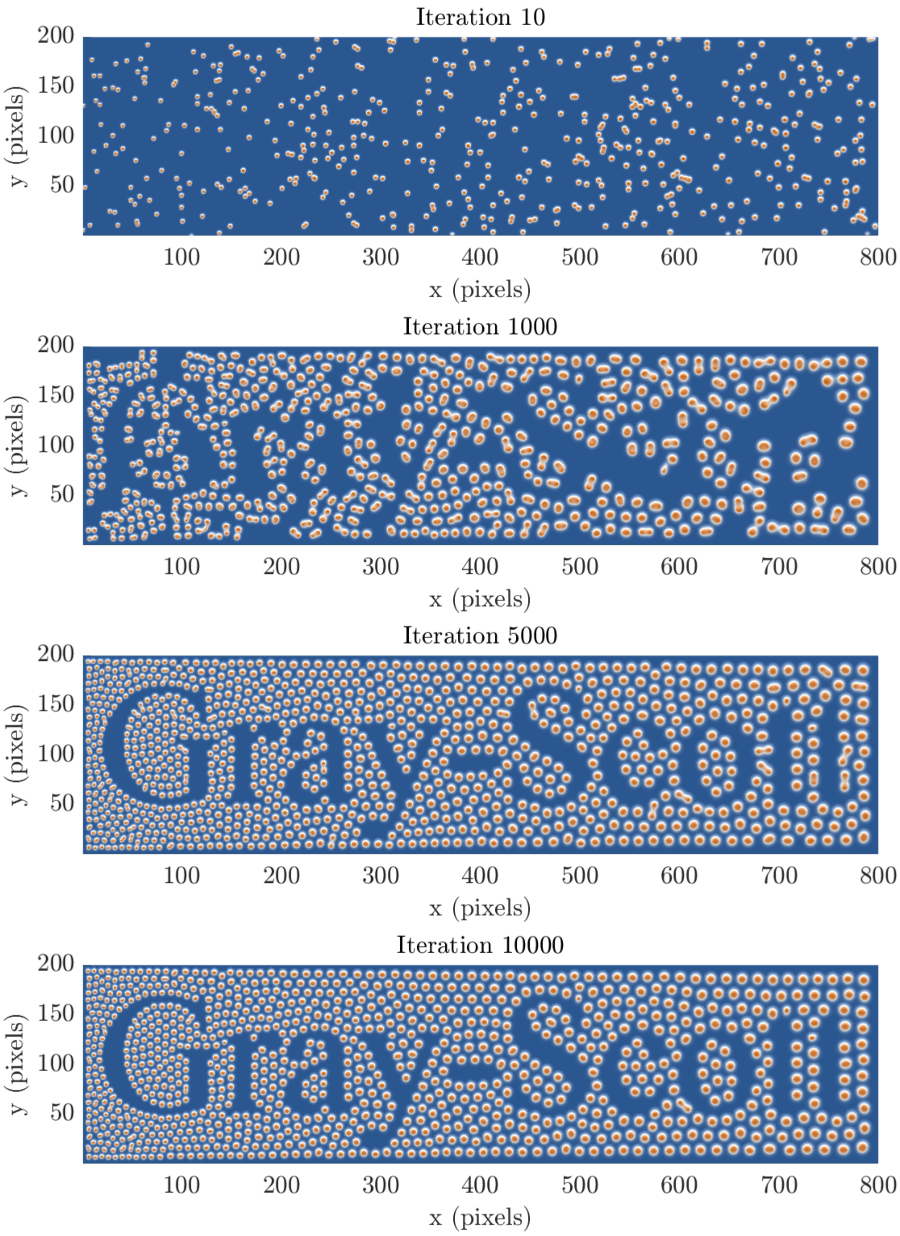

Figure S4: Growth and structuring of morphogens in a domain of 800 by 200 pixels. A cell pattern is selected by imposing everywhere a couple of parameters $(f, k) = (0.0325, 0.063)$, except on a set of pixels forming the "Gray-Scott" names where the values are $(f, k) = (0, 0.063)$. The diffusion constant $\mathbf{d_u}$ is modulated linearly from left to right, from a value of 0.2 to 1 in order to control the dimensions of the generated patterns. The parameter $\mathbf{d_v}$ is determined for the whole space such that $\mathbf{d_u} = \mathbf{d_v}/2$.

tensors, it is possible to make a geometrical representation of the latter to facilitate their manipulation (Fig. S5).

Following the characterization of the patterns generated in the rest of this paper, a direct correspondence between the eigenvectors of the diffusion tensor and those of the generated reactance tensors allows the following diagonalization:

$$\underline{\underline{\mathbf{D}}} = \underline{\underline{\mathbf{R}}}_{\phi}\, \text{diag}(\boldsymbol{\lambda}_1, \boldsymbol{\lambda}_2)\, \underline{\underline{\mathbf{R}}}_{\phi}^{T}, \tag{27}$$

where $\boldsymbol{\lambda}_1$ and $\boldsymbol{\lambda}_2$ correspond to the eigenvalues of the diffusion tensors $\underline{\underline{\mathbf{D}}}$ and where $\underline{\underline{\mathbf{R}}}_{\phi}$ remain the same rotation matrices as previously defined, containing local eigenvectors.

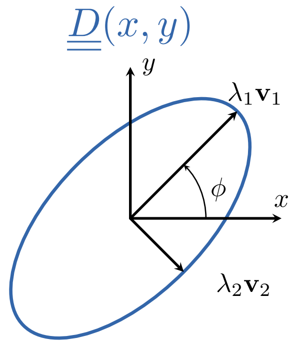

Figure S5: Representation of a diffusion tensor by means of an ellipse whose principal axes correspond to the eigenvectors of the tensor, weighted by the associated eigenvalues. These axes help to visualize the anisotropy and the orientation imposed for the diffusion of morphogens.

The numerical implementation of this anisotropic diffusion can again be computed by finite differences. In this new situation, different weights are applied to the various elements of the discrete Laplacian operator in order to create privileged directions for the diffusion. Adapting the work of Witkin and Kass for the synthesis of computational textures [10], we obtain the following mask:

$$L = \begin{bmatrix} -D_{xy} & 2D_{yy} & D_{xy} \\ 2D_{xx} & -4\text{Tr}(\underline{\underline{\mathbf{D}}}) & 2D_{xx} \\ D_{xy} & 2D_{yy} & -D_{xy} \end{bmatrix}, \tag{28}$$

where the different elements of the diffusion tensor are determined at each point of the metasurface according to the associated eigenvalues (λ_1, λ_2) and the orientation of the

principal axis ϕ:

$$D_{xx} = \left(\lambda_1 \cos^2(\phi) + \lambda_2 \sin^2(\phi)\right) \tag{29}$$

$$D_{yy} = \left(\lambda_2 \cos^2(\phi) + \lambda_1 \sin^2(\phi)\right) \tag{30}$$

$$D_{xy} = (\lambda_1 - \lambda_2)(\cos(\phi)\sin(\phi)) \tag{31}$$

$$D_{yx} = -D_{xy}. \tag{32}$$

The conservation of morphogens is ensured by normalizing the trace of the diffusion tensor, implying that $\mathrm{Tr}(\underline{\mathbf{D}}) = \lambda_1 + \lambda_2 = 1$. Under these conditions, it is possible to simplify the expression of the anisotropic Laplacian operator:

$$L = \begin{bmatrix} -D_{xy}/4 & D_{yy}/2 & D_{xy}/4 \\ D_{xx}/2 & -1 & D_{xx}/2 \\ D_{xy}/4 & D_{yy}/2 & -D_{xy}/4 \end{bmatrix} \tag{33}$$

The implementation of this new model now guarantees a local control the orientation of the generated Turing patterns (Fig. S6).

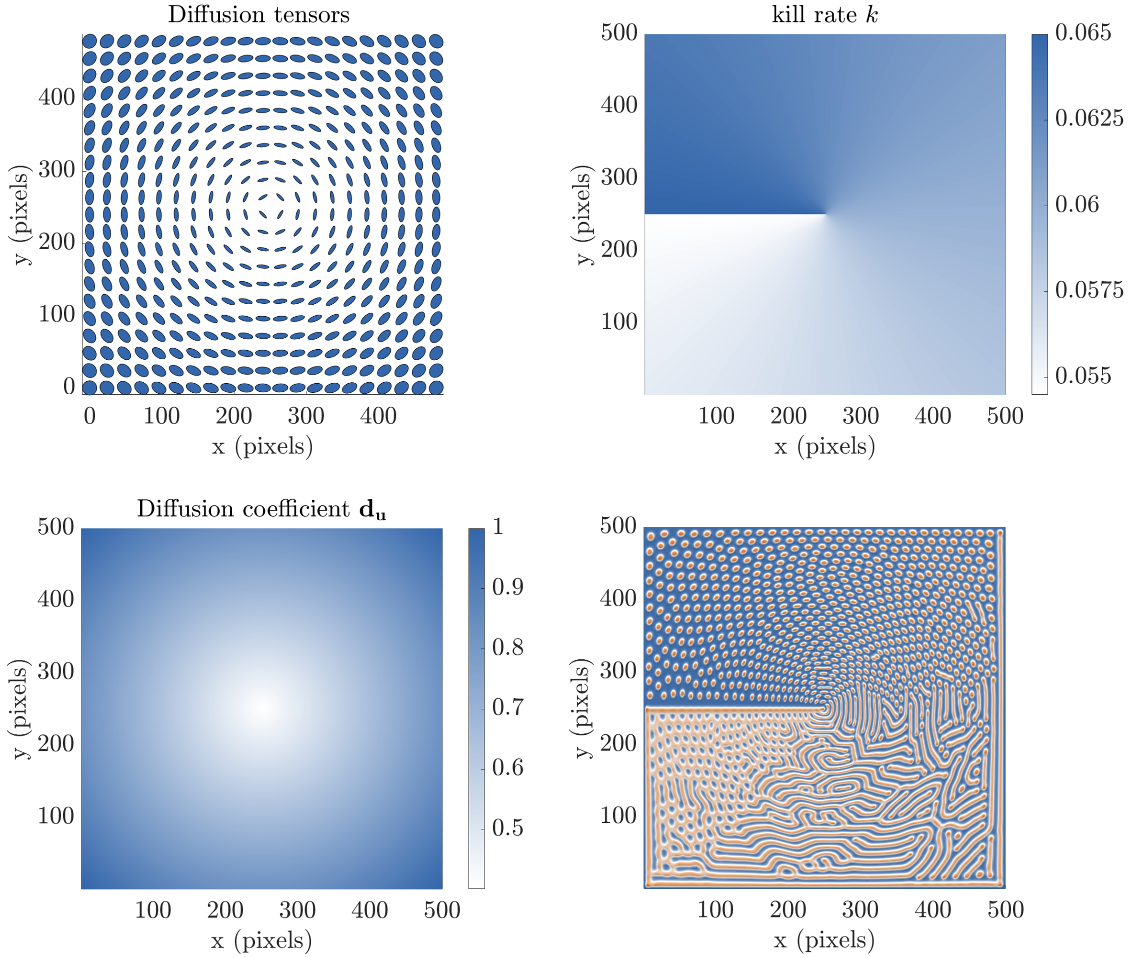

Figure S6: Anisotropic generation based on the modified Gray-Scott model. A sample of the diffusion tensors is represented, varying radially from anisotropic to isotropic distributions. An azimuthal variation of the kill rate k defines the type of generated patterns and the associated feed rate f is kept constant such that $f = 0.03$. The diffusion coefficient $\mathbf{d_u}$ is modulated radially between 0.4 and 1, impacting the local dimensions of the synthesized shapes. The parameter $\mathbf{d_v}$ is finally determined for the whole space such that $\mathbf{d_v} = \mathbf{d_u}/2$.

This anisotropic generation also highlights the unequal response of different types of Turing patterns to the diffusion tensors, possibly justified by the dominance of reaction terms over the diffusion process. Following the definition of a generative model allowing the automated synthesis of anisotropic patterns, it is then necessary to characterize the electromagnetic properties associated with the generated shapes.

3 Electromagnetic characterization of the anisotropic patterns

The characterization of the electromagnetic properties synthesized using this generative model is presented in this section. Cellular patterns are generated by imposing a pair of parameters $(f, k) = (0.032, 0.063)$ for the rest of this work. Diffusion constants $d_u = 0.95$ and $d_v = d_u/2$ are also fixed. Following a first phase of characterization, the patterns are finally generated using the anisotropic model on a grid of 68×68 pixels for a super cell dimension of 13 mm side. These dimensions are chosen in order to reach an average surface reactance of 270Ω, impacting the spatial frequencies of the printed distributions. These dimensions also guarantee that the gaps between the cellular patterns remain compatible with chemical etching technologies. Finally, the patterns are generated to present periodic boundary conditions, facilitating the simulation of infinite media without geometric breaks. This continuity is ensured by the exploitation of the discrete Laplacian operator, applied with a spatially periodic convolution product.

Two degrees of freedom are chosen to modulate the properties of the generated reactance tensors. On the one hand, the largest eigenvalue of the diffusion tensor λ_1 is used to determine the anisotropy of the generated patterns. We recall that by normalizing the trace of the diffusion tensor, we impose $\lambda_2 = 1 - \lambda_1$, thus reducing the number of variables in the problem. A second degree of freedom is introduced by performing a thresholding of the Turing patterns, allowing to adjust the gaps between the elliptic shapes. Following an upscaling of a factor 4 and an amplitude normalization of the generated patterns for the morphogens $\mathbf{V}$, only the pixels satisfying the inequation $|\mathbf{V} - \sigma + 0.01| > \sigma$ are retained. Among all the possible choices offering the control of the synthesized electromagnetic properties, a simple thresholding helps to accelerate the characterization phase of the synthesized properties by limiting the number of generations to the variations of λ_1. The addition of a supplementary threshold with a value of 0.01 avoids the appearance of a coalescence phenomenon between close patterns, especially when the threshold is at its lowest values. For the same reasons, we impose a limitation of the eigenvalues of the diffusion coefficients such as $\lambda_1 \in [0.15, 0.85]$, thresholds beyond which the generated elliptical patterns tend to locally merge under the action of a large diffusion coefficients. An example of generation is presented in Fig. S7.

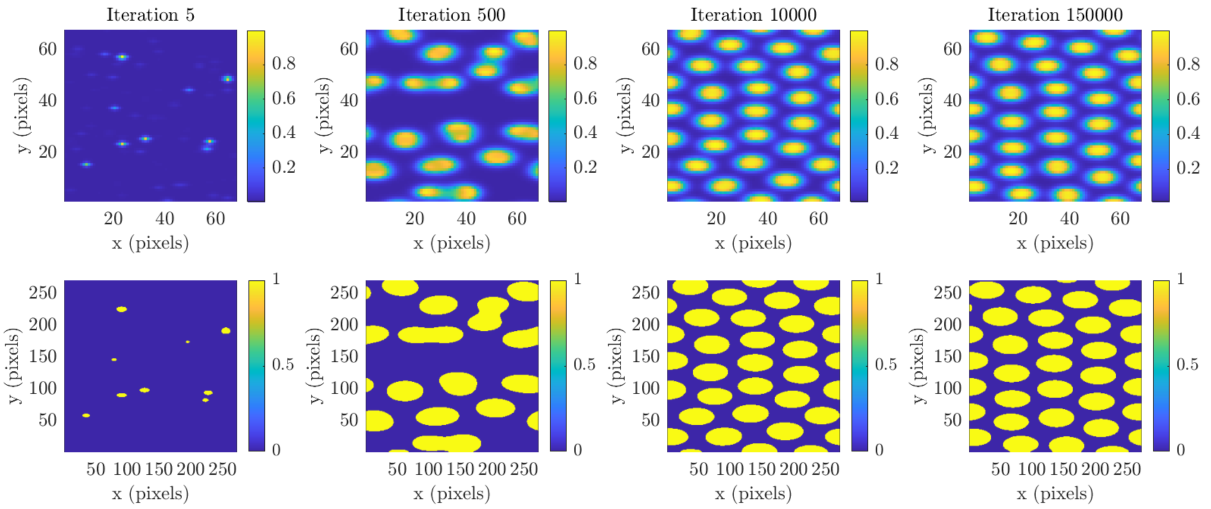

Figure S7: Generation of a periodic pattern allowing the characterization of the synthesized reactance properties. All the iterations are computed in 14 seconds, including the time associated to the export of the figures. The top figures correspond to the patterns generated for different iterations and the bottom ones are the equivalent versions after upscaling by a factor of 4 and thresholding. The yellow parts correspond to the copper elements on the upper side of the metasurface while the blue areas are the etched parts, revealing the dielectric of the considered substrate.

Following the generation of a pattern for a set of parameters, it is then possible to export the obtained geometry to the surface of a dielectric, forming metallic elliptical grains allowing the synthesis of reactance tensors by a homogenization effect. The simulations are performed on a Rogers RO3003 substrate with a relative permittivity $\epsilon_{r_1} = 3$, a loss tangent $\tan(\delta) = 10^{-3}$ and a thickness $h = 1.52$ mm. This substrate is chosen to facilitate the fabrication of proofs of concept, exploiting the relatively low permittivity to limit the dispersion of the anisotropy properties and the dimensions of the patterns to be etched. On the other hand, a low permittivity implies a reduced ability to modulate the anisotropy of the synthesized tensors. The exported patterns can finally be characterized by electromagnetic simulation using a finite element method in the frequency domain, implemented using the commercial software *CST Studio Suite*. By imposing periodic boundary conditions compatible with the constraints imposed during the generation of the patterns, an infinite medium can finally be characterized by means of a Floquet port exciting two orthogonal plane waves in normal incidence (Fig. S8).

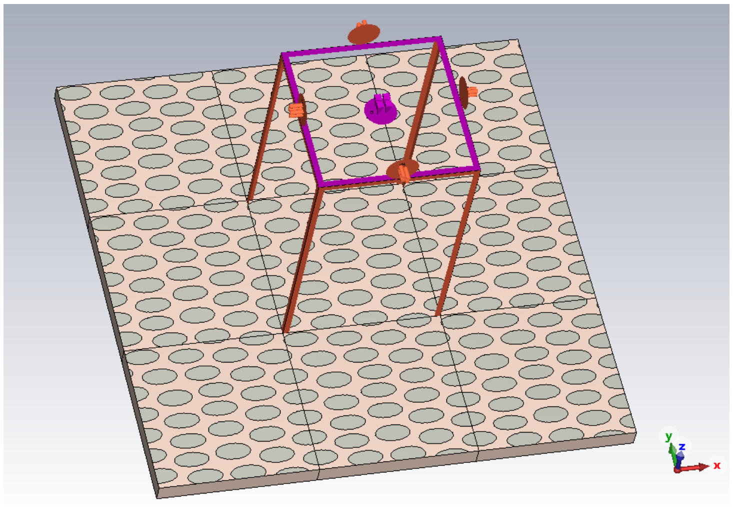

Figure S8: Finite Element Method (FEM) simulation of a pattern placed under periodic boundary conditions using *CST Studio Suite*.

These simulations are used to extract 4 complex terms of scattering parameters for each simulated frequency, corresponding to the interaction between two orthogonal transverse electromagnetic excitations in emission and reception oriented according to the reference axes imposed by the simulation. The phase associated with the back and forth propagation in the air is compensated by de-embedding the Floquet port. This correction finally allows the reconstruction of the associated impedance matrix under plane wave excitation, noted η_{PW}. The diagonalization of the obtained impedance tensors reveals a direct correspondence between the ϕ orientation of their principal eigenvector and that of the diffusion tensor used for the generation of the Turing patterns. Although intuitive, this validation confirms that it is possible to limit the characterization of the electromagnetic properties synthesized with patterns to the modulation of the anisotropy.

It is important to note at this point that the impedance reconstructed under these illumination conditions differs from that encountered by the transverse magnetic excitation that we will use later. A conversion method proposed by Patel and Grbic [13] is thus exploited to determine the impedance tensor (and thus the reactance for its imaginary part only) directly useful for the conversion of surface waves into radiated waves. The impedance η_{PW} determined from a plane wave illumination at normal incidence corresponds to the parallel contribution of the surface formed by the Turing patterns η_{sheet} and of the propagation within the dielectric, modeled according to the transmission line theory:

$$\frac{1}{\eta_{\mathrm{PW}}} = \frac{1}{\eta_{\mathrm{sheet}}} + \frac{1}{j\frac{\eta_0}{\sqrt{\epsilon_{r_1}}}\tan(k_0\sqrt{\epsilon_{r_1}}h)}. \tag{34}$$

In this expression, η_0 and k_0 correspond to the characteristic impedance and the wave number of free space. It is thus directly possible to determine η_{sheet} from the electromagnetic properties of the dielectric. Having removed the contribution of the plane wave within the dielectric, it is now necessary to add that of the transverse magnetic wave which will soon be excited by the monopole placed within the metasurface, corresponding to a new term in parallel as illustrated by Fig. S9.

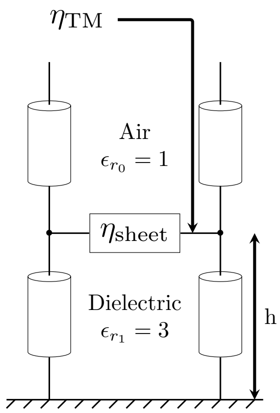

Figure S9: Transmission line model highlighting the contribution of the dielectric and the surface formed by the Turing patterns in the constitution of the impedance seen by a magnetic transverse excitation.

In these conditions, the determination of the impedance η_{TM} is more delicate because its expression intervenes in two places in the dispersion of relation of the modes within the structure [13]:

$$\frac{1}{\eta_{\text{TM}}} = \frac{1}{\eta_{\text{sheet}}} + \frac{1}{j\frac{\eta_0\, k_{x_1}}{k_0\, \epsilon_{r_1}} \tan(k_{x_1} h)}, \tag{35}$$

where

$$k_{x_1} = \sqrt{k_0^2(\epsilon_{r_1} - 1) + \left(\frac{\eta_{\text{TM}}\, k_0}{\eta_0}\right)^2}. \tag{36}$$

For each simulation, it is thus necessary to solve this problem numerically to determine the value of η_{TM}.

The studied problem being tensorial, this method must be applied for the different components of the impedance tensors η_{PW} initially determined. To limit the number of computations, these transformations are performed on the eigenvalues of these tensors only, revealing the impedance of the patterns alone and then the one seen by an incident

TM mode according to the eigendirections imposed by the diffusion tensors, in connection with Eq. (11).

Following a series of generations varying the parameters λ_1 and σ previously introduced, it is finally possible to characterize the reactances X_1 and X_2 according to the main directions imposed for each pattern (Fig. S10).

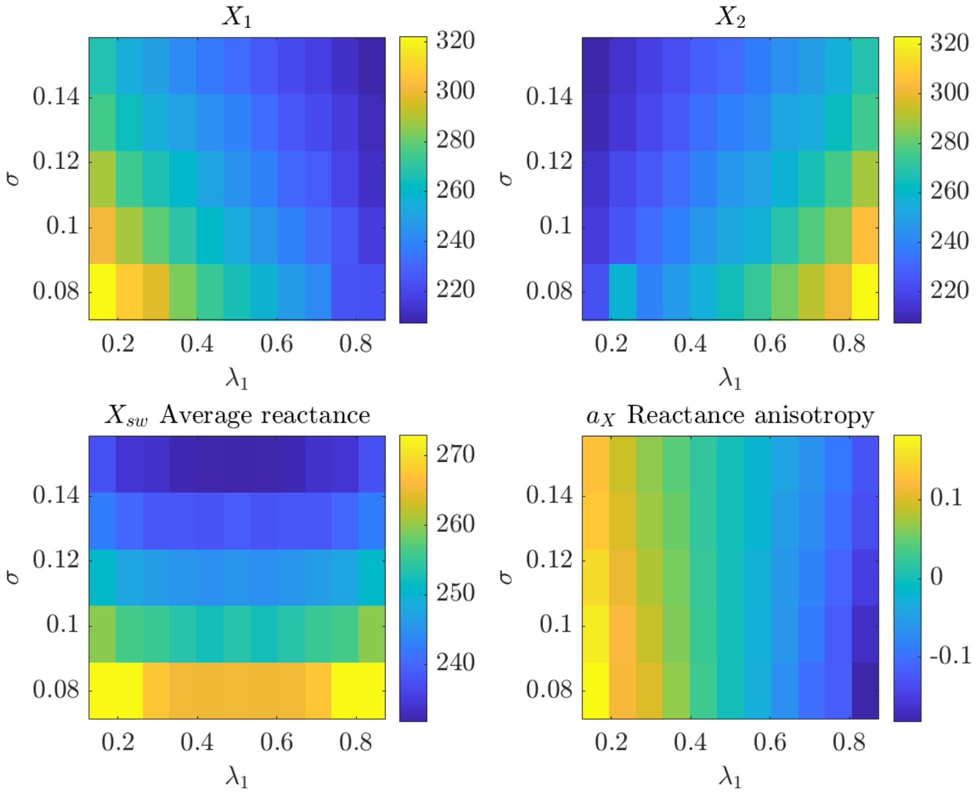

Figure S10: Electromagnetic characteristics determined for a series of simulations from patterns generated by varying the anisotropy through the eigenvalue λ_1 of the diffusion tensor and by changing the thresholding of the patterns by means of the parameter σ.

To facilitate the interpretation and processing of these results, the reactance values obtained along the principal directions of the tensors are transformed into the average reactance seen by the surface wave X_{sw} and the anisotropy modulation coefficient a_X, respectively determined as:

$$X_{sw} = \frac{X_1 + X_2}{2} \tag{37}$$

$$a_X = \frac{|X_1 - X_{sw}|}{X_{sw}} \tag{38}$$

In this way, the modulation of the reactance values can also be expressed to correspond to Eq. s (13) and (14):

$$X_1 = X_{sw}(1 + a_X) \tag{39}$$
$$X_2 = X_{sw}(1 - a_X) \tag{40}$$

The obtained parameters are the result of a homogenization process that can be improved by averaging. Exploiting the symmetries of the problem, it is first proposed to consider that the generated tensors are isotropic for $\lambda_1 = \lambda_2 = 0.5$ and that a positive or negative deviation around this value will generate the same reactance tensor with a rotation of 90° around, producing identical mean reactances X_{sw}. The obtained results can thus be averaged by symmetry around the axis $\lambda_1 = 0.5$. For similar reasons, the anisotropy modulation coefficients a_X should be identical, with the opposite sign, around the value $\lambda_1 = 0.5$ for which we will always obtain $a_X = 0$. Following the identification of a range of parameters that allow mean values of X_{sw} to be reached between 250Ω and 280Ω, i.e. $\lambda_1 \in [0.19, 0.81]$ and $\sigma \in [0.07, 0.1]$, a characterization less dependent on the random initialization conditions of the patterns is achieved by averaging the results of 10 simulations for each pair of parameters. To ensure the local generation of the desired electromagnetic characteristics on a metasurface, it is finally necessary to determine the parameters (λ_1, σ) generating each couple of values (X_{sw}, a_X). This parameter-fitting is carried out for a mean reactance of $X_{sw} = 270\Omega$, the set of solutions of which are represented in Fig. S11.

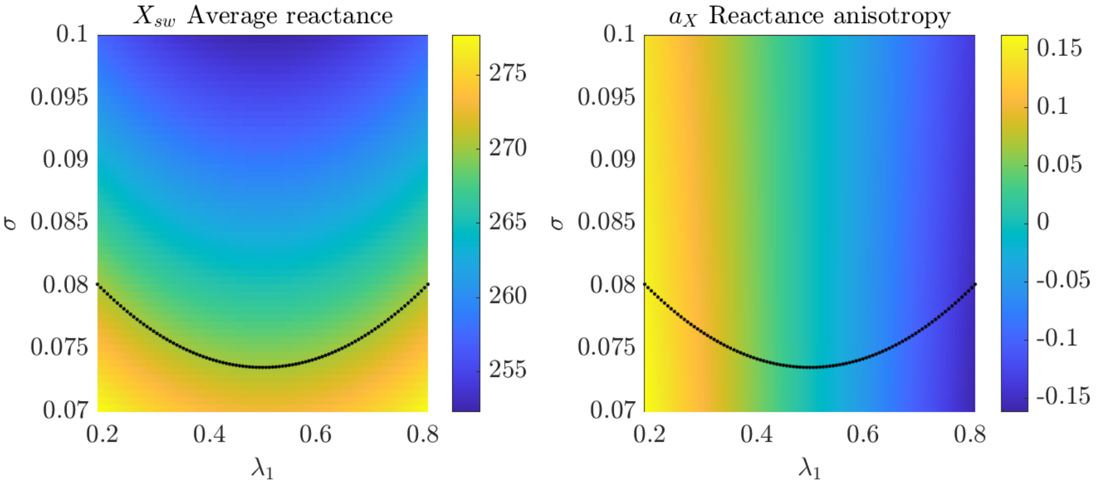

Figure S11: Averaged characteristics linking the generation parameters (λ_1, σ) to the synthesized electromagnetic properties. The set of points associated with an average reactance of $X_{sw} = 270\Omega$ are represented.

To simplify the generation of the desired properties, a quadratic model is adapted to impose a relation between the coefficients λ_1 and σ that maintain an average reactance

$X_{sw} = 270\Omega$:

$$\sigma(\lambda_1) = p_{\sigma_2}\lambda_1^2 + p_{\sigma_1}\lambda_1 + p_{\sigma_0} \tag{41}$$

In this model and under the considered conditions, the coefficients are respectively $p_{\sigma_2} = 0.06893$, $p_{\sigma_1} = -0.06893$ and $p_{\sigma_0} = 0.09079$. Now having a link between these two generation parameters, it is only necessary to set the value of λ_1 that allows the desired anisotropy modulation coefficient a_X to be reached. Following the extraction of a_X values along the path indicated in Fig. S11, a linear fit is now proposed to model the relationship between these two parameters, as introduced in the following equation and in Fig. S12.

$$a_X(\lambda_1) = p_{a_1}\lambda_1 + p_{a_0} \tag{42}$$

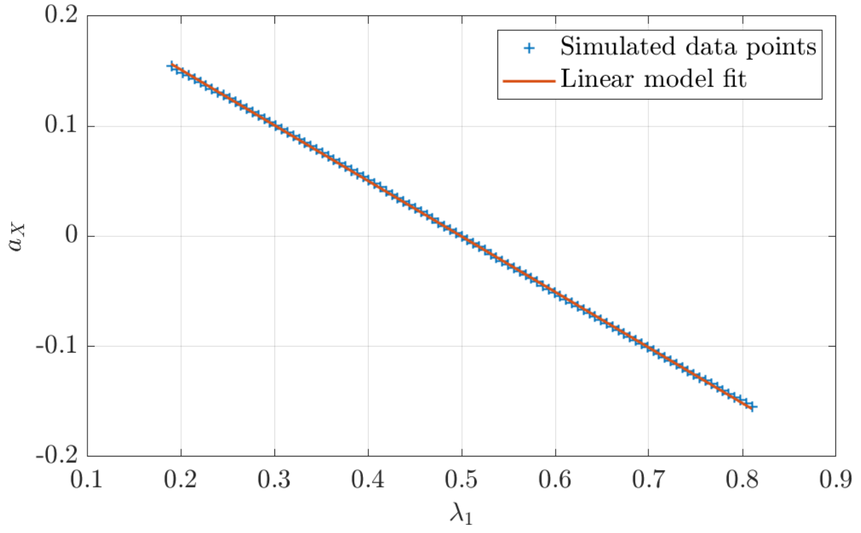

Figure S12: Evolution of the anisotropy modulation coefficient a_X as a function of the parameter λ_1, corresponding to the main eigenvalue of the diffusion tensor guiding the generation of the Turing patterns. A simple linear fit is performed on these extracted data at constant mean reactance.

The good correspondence of the linear model with the data from simulation greatly simplifies the synthesis of the expected properties. This generation can indeed be performed at the scale of metasurfaces with large dimensions in front of the operating wavelengths, imposing a very large number of morphogenetic parameters to be determined on the whole surface to satisfy local electromagnetic constraints. Obtaining a particularly simple analytical model between an anisotropy modulation coefficient a_X and the associated generation properties thus avoids the need for computationally expensive iterative methods that would be necessary at the scale of all the pixels composing the metasurface.

In the considered case, the anisotropy of the diffusion tensor can finally be calculated as follows:

$$\lambda_1 = \frac{a_X(\lambda_1) - p_{a_0}}{p_{a_1}}, \tag{43}$$

recalling that we then determine the second eigenvalue such that $\lambda_2 = 1 - \lambda_1$ and that the eigenvectors are the same as those of the objective reactance tensor. The values obtained for this last model are $p_{a_1} = -0.505$ and $p_{a_0} = 0.2525$.

In summary of the synthesis steps, it is first necessary to define an objective electric field $\mathbf{E}_t|_{z=0^+}$ in the radiating aperture allowing to satisfy far field or near field radiation conditions. An objective reactance tensor $\underline{\underline{\mathbf{X}}}$ can then be determined by Eq. (10). The reactance modulation of the latter is adapted by choosing a constant value $a_X^{\max}$ according to the achievable ones for a chosen substrate type and pattern dimensions. At each point of the metasurface, the objective tensors must be diagonalized to reveal the reactances $\mathbf{X}_1$ and $\mathbf{X}_2$ according to the main directions, enabling the computation of the anisotropy modulation coefficient $a_X \in [-a_X^{\max}, a_X^{\max}]$. The eigenvectors resulting from Eq. (12) are directly re-used for the definition of the diffusion tensors following Eq. (27). Determining a constant reactance on the whole surface, it is then possible to compute the eigenvalues of the diffusion tensor according to the anisotropy of the desired reactance tensors by Eq. (43), and then to deduce the associated local thresholding of the Turing patterns through Eq. (41). The set of parameters required for the growth of a morphogenetic metasurface are at this stage defined and the iteration of the anisotropic Gray-Scott model then converge towards a structuring ensuring an effective conversion of the surface waves into radiation objectives.

4 Parameters used in this work

The parameters presented in the different numerical models introduced in this work are gathered in the following table to facilitate the reproduction of these results:

Kill rate k	0.063
Feed rate f	0.032
Diffusion constant d_u	0.95
Diffusion constant d_v	$d_u/2$
Average reactance X_{sw}	270Ω
Maximum reactance modulation $a_X^{\max}$	0.186
Magnitude-based modulation α	0.5
Pixels per side	1196
Spatial resolution	0.191 mm
Dimensions of the metasurfaces	229×229 mm^2
Substrate Rogers RO3003	$\epsilon_{r_1} = 3$ - $\tan(\delta) = 10^{-3}$ - h = 1.52 mm

Supplementary Table 1: List of parameters considered in this work

The spatial resolution and the number of pixels per side correspond to the reaction-diffusion generation parameters. The optimization of the latter was performed during the characterization phase of the generated patterns, defining their dimensions and electromagnetic characteristics. This operation only needs to be performed once for a given substrate and frequency band, enabling the synthesis of controlled reactance tensors for any desired metasurfaces.

Morphogens are initialized everywhere such that $\mathbf{U} = 1$ and $\mathbf{V} = 0$. The reaction-diffusion is then triggered by randomly passing a few pixels of $\mathbf{V} = 0.7$.

The generations are all achieved in 100 000 iterations, performed with a *Matlab* code for the dimensions presented in less than 7 minutes on a computer equipped with a CPU i9-10900K running at 3.70GHz.

In order to limit the staircase effects on the edges of the generated geometries, the morphogen matrices are interpolated by a factor of 4 before their export.

Two adjustments of the model were made to reach the presented performances with the help of a series of electromagnetic simulations carried out on models of reduced dimensions. On the one hand, the wave number was corrected upwards following the achievement of

maximum gain at frequencies slightly lower than those anticipated:

$$\beta_{sw} = 1.0089\, k_0 \sqrt{1 + \left(\frac{X_{sw}}{\eta_0}\right)^2} \tag{44}$$

This correction, deduced from simulations and linked to the considered approximations, might be lowered to provide a correction of the frequency shifts observed in measurement.

A second modification concerns this time only the single-polarized far-field metasurface presented in this work. The anisotropy modulation tends to create flattened elliptical patterns for the farthest regions of the monopole, especially in the case of this metasurface where the orientation distribution of the diffusion tensors is the most uniform due to the single radiation objective. Approaching the anisotropy limits of the diffusion tensors in the case studied, the cells can tend to coalesce at certain points, creating larger patterns forming local impedance discontinuities that can hinder the performance of the metasurfaces. To limit this phenomenon of local merging between cells, the kill rate k has been slightly increased such that $k = 0.0635$ instead of $k = 0.063$ used for the two other metasurfaces and in the characterized model. The performances were thus slightly improved, reaching the values predicted by a numerical model based on the definition of surface reactances.

The impedance matching of the monopole exciting the surface wave was performed by etching a simple pattern whose dimensions are given in Fig. S13. This design is notably inspired by the numerous contributions of the research group of the University of Siena led by Stefano Maci [4, 14].

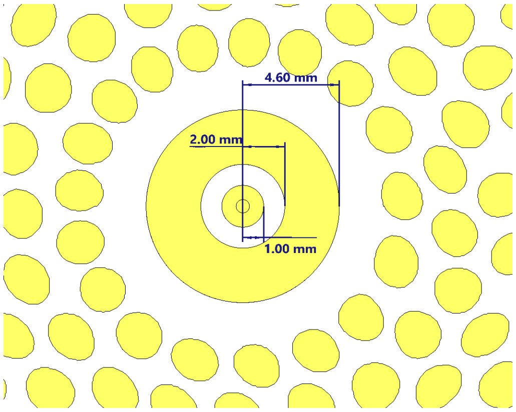

Figure S13: Geometry of the monopole facilitating the matching to the average surface impedance presented by the metasurface.

The coaxial connector used for these three metasurfaces is the reference 1012-24SF made by the company *Southwest*.

The metasurfaces synthesized for the two far field experimental demonstrations are finally presented in Fig. S14.

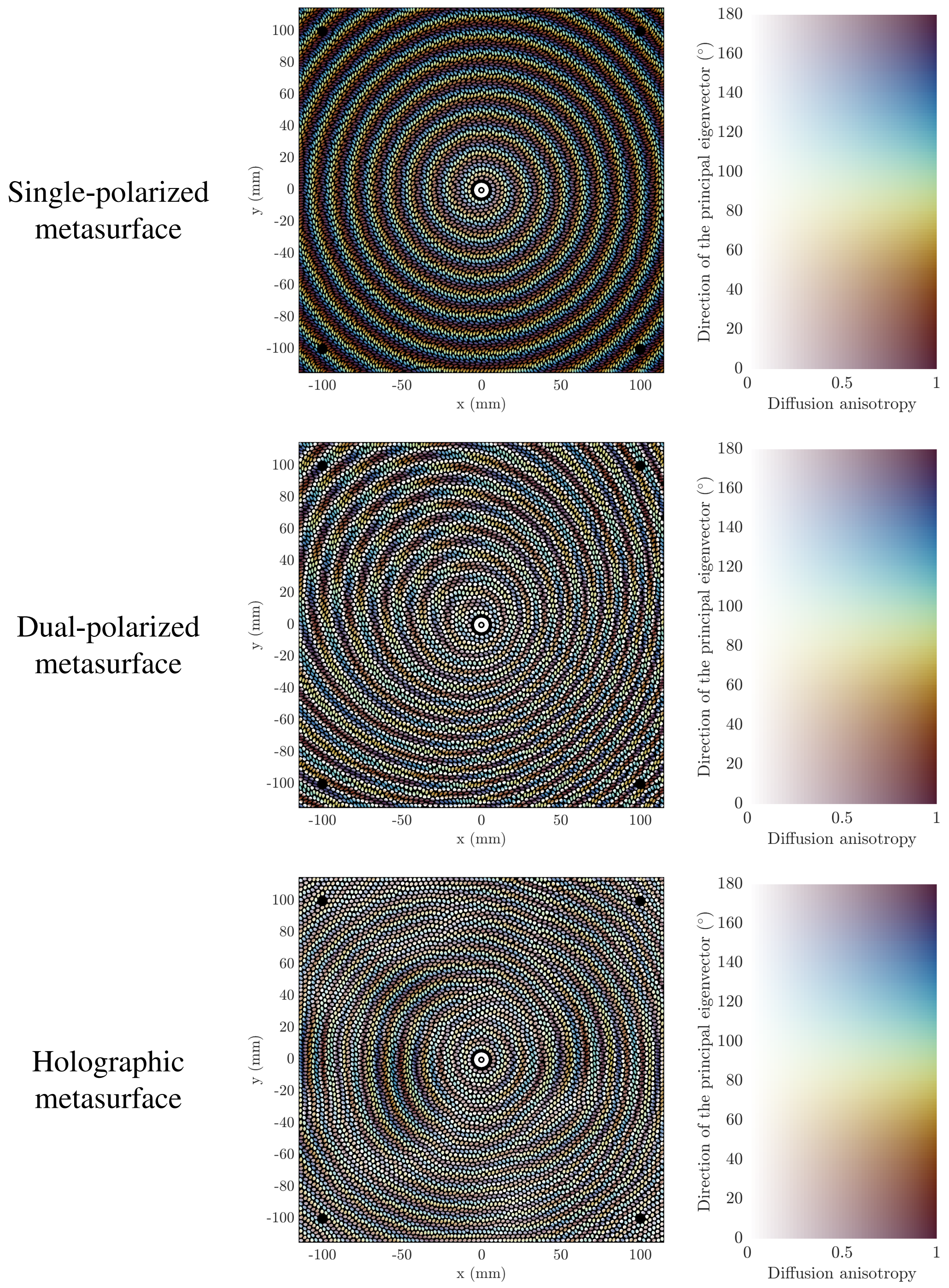

Figure S14: Morphogenetic metasurfaces generated, fabricated and measured in this work. The colormap represents the orientation of the principal vector (color) and the anisotropy of the diffusion tensors (saturation) used for morphogen growth.

5 Measurement Techniques

Realized gain measurements are performed around 20 GHz in an anechoic chamber. Far field conditions are obtained using a parabolic reflector and allow the measurement of two orthogonal polarizations (Fig. S15). Numerical processing is then applied to convert this data into circular polarizations. The uncertainty of gain measurement is evaluated at ±0.6 dB.

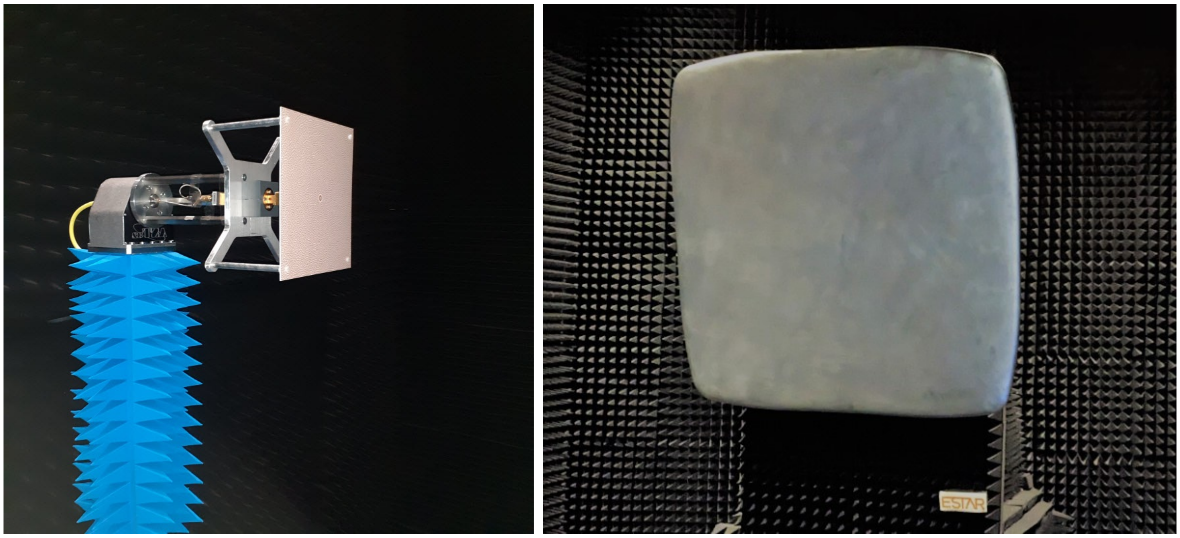

Figure S15: Far field radiation measurement. (**Left**) Metasurface mounted on a support rotating along two axis. (**Right**) Parabolic reflector used to convert the spherical wavefront of a reference antenna in plane wave.

The near field measurements are performed using a Cartesian scanner equipped with an open-ended waveguide designed for the K band (18-26.5 GHz). A scan is performed in a plane parallel to the radiating aperture at a distance of 20 cm from the latter, according to an identical stroke for each axis of a total length of 275 mm sampled every 5 mm (or 51 points per axis). The field is measured successively according to the vertical and horizontal polarizations by rotating the waveguide, allowing the data to be represented with circular polarizations by linear combinations of the measurements.

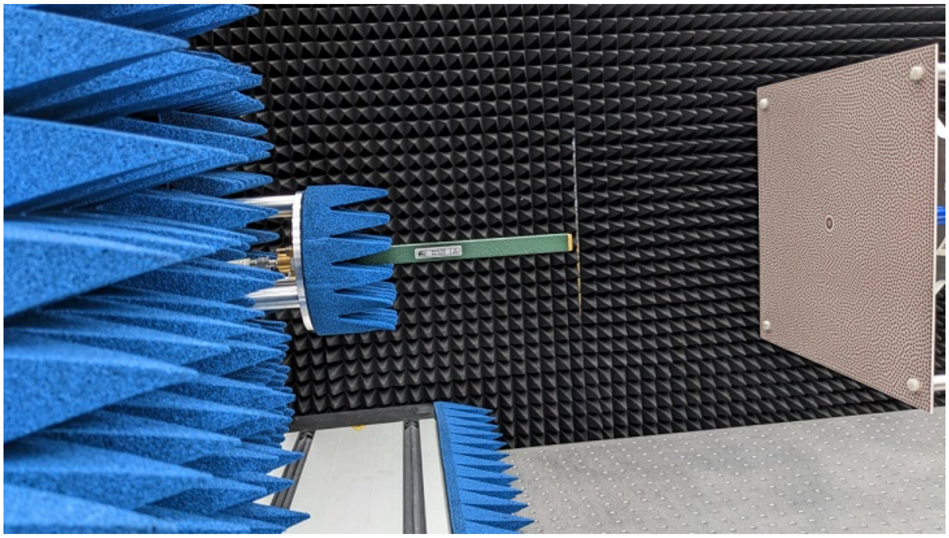

Figure S16: Cartesian scanner equipped with a reference antenna for measuring the radiated near field.

6 Supplementary experimental results

High gain LHCP metasurface antenna

Measurement results are performed in the left-hand circularly polarized beam axis ($\theta = 0°$ and $\phi = 0°$) and summarized in Fig. S17. A maximum gain of 31.3 dBi is measured for the frequency f = 20.3 GHz. The half-power bandwidth (HPBW) is 1.25 GHz, for a center frequency of 20.2 GHz, or a relative bandwidth of 6.4%. A slight dissymmetry of the gain is at the origin of the small shift between the frequency of the maximum value and that of the center of the band but the variations between these two points do not exceed 0.05 dB.

Following the measurement of 3D diagrams for 5 frequency points centered around 20.3 GHz and spaced by 0.5 GHz, directivities are calculated and displayed on the same figure. These markers provide an estimate of the total loss level. The metasurface antenna technology is particularly competitive in these aspects, offering in the proposed case a maximum directivity of 31.7 dBi, which is only 0.4 dB higher than the measured gain. This difference tends to increase when deviating from the initial frequency of operation of the antenna, reaching 1.7dB at the frequency of 21.3 GHz.

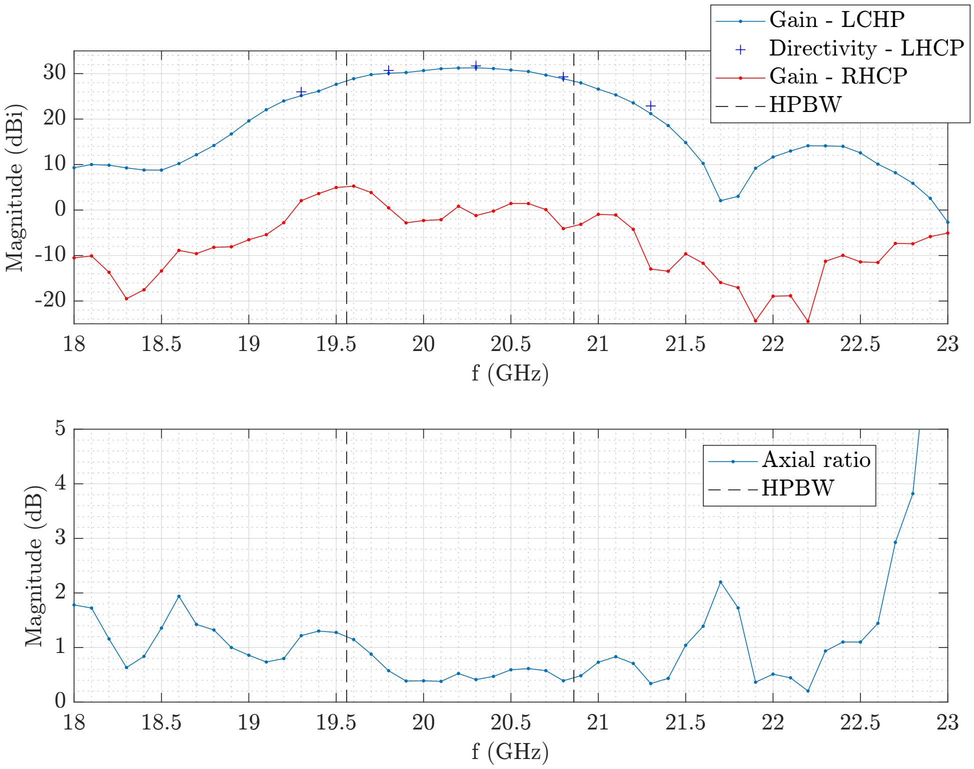

Figure S17: Frequency measurements of gain and directivity in the LHCP beam axis.

The gains realized in left and right circular polarizations are represented in 3D at 20.3 GHz in Fig. S18.

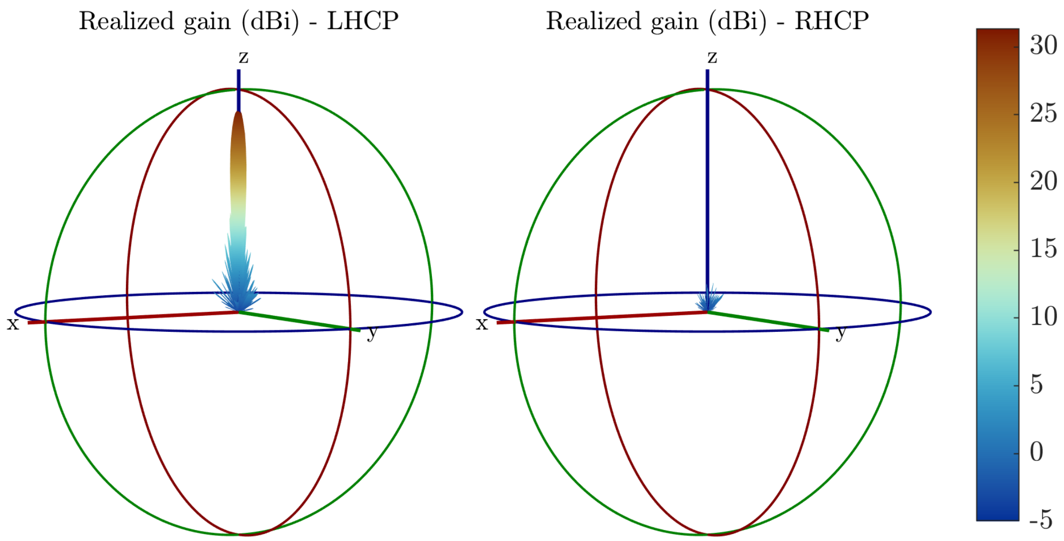

Figure S18: Realized gains measured at 20.3 GHz.

Dual polar metasurface antenna

Measurement results are performed in the left-hand circularly polarized beam axis ($\theta = -29.5°$ and $\phi = 0°$) and summarized in Fig. S19. A maximum gain of 25.4 dBi is measured for the frequency f = 20.2 GHz. The half-power bandwidth (HPBW) is 1.15 GHz, for a center frequency of 20.2 GHz, or a relative bandwidth of 5.7%.

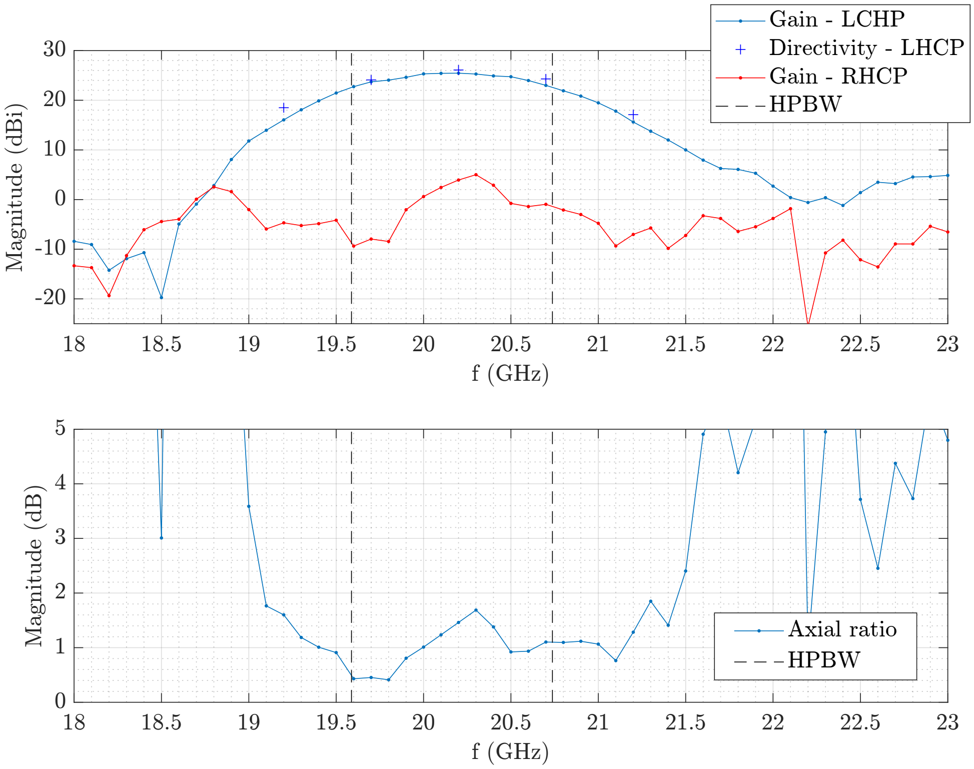

Figure S19: Frequency measurements of gain and directivity in the LHCP beam axis.

A directivity of 26.1 dBi is determined at the same frequency from the three-dimensional measurement of radiation patterns, i.e. a deviation from the realized gain of 0.7dB due to losses.

A comparable analysis is performed for the beam formed in right-hand circular polarization (Fig. S20). Comparable results are obtained for this second lobe in a direction $\theta = 29.5°$ and $\phi = 90°$. The maximum gain extracted from the three-dimensional measurements is 25.2 dBi at 20.2 GHz, while the estimated directivity at the same frequency is 25.9 dBi, again a difference of 0.7dB.

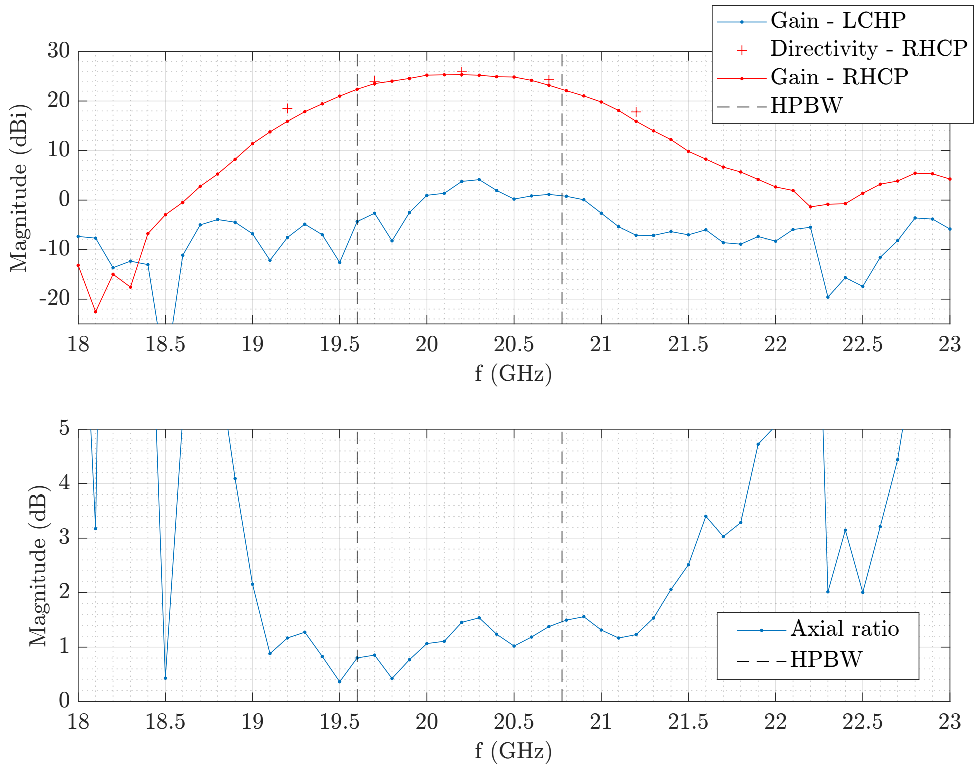

Figure S20: Frequency measurements of gain and directivity in the RHCP beam axis.

The measured gains in left and right-hand circular polarizations, revealing the multiplexing of the two beams, are finally presented in Fig. S21.

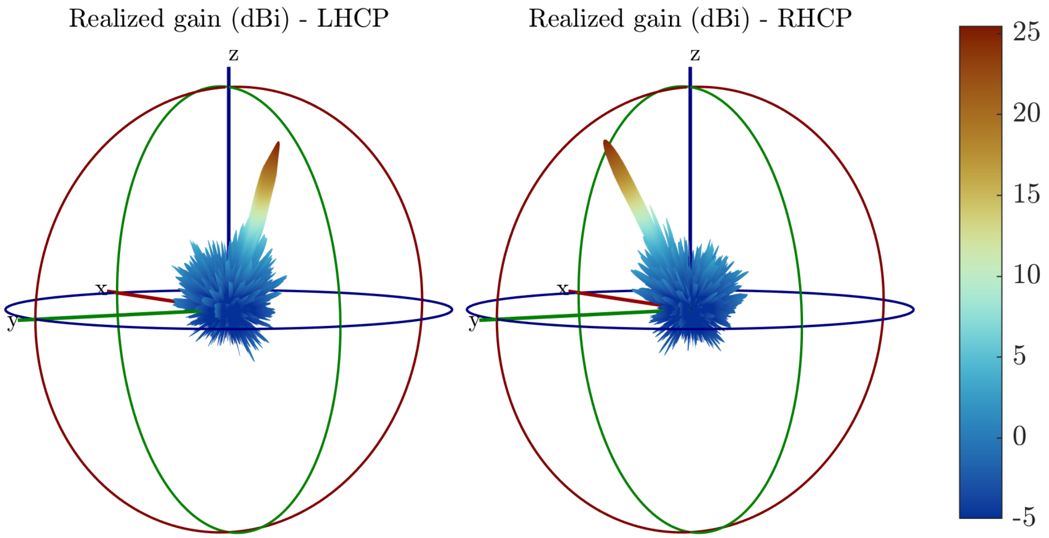

Figure S21: Realized gains measured at 20.2 GHz.

Near-field holography

A morphogenetic metasurface is finally designed to radiate a dual circularly polarized electromagnetic field in the radiative near-field (Fresnel) zone following a holographic principle. The objective is to generate in left-hand circular polarization the letter "L", and in right-hand circular polarization the letter "R" while obtaining a weak coupling between these two spatial distributions. Following the procedural synthesis and fabrication of this square metasurface of 229 mm side, the field is measured in a plane parallel to the radiating aperture at a distance of 200 mm. The scanned area covers 275 by 275 mm^2 sampled every 5.5 mm along both axes. To facilitate visualization of the distances involved in this experiment, the metasurface is shown facing the field scans measured in circular polarization in Fig. S22.

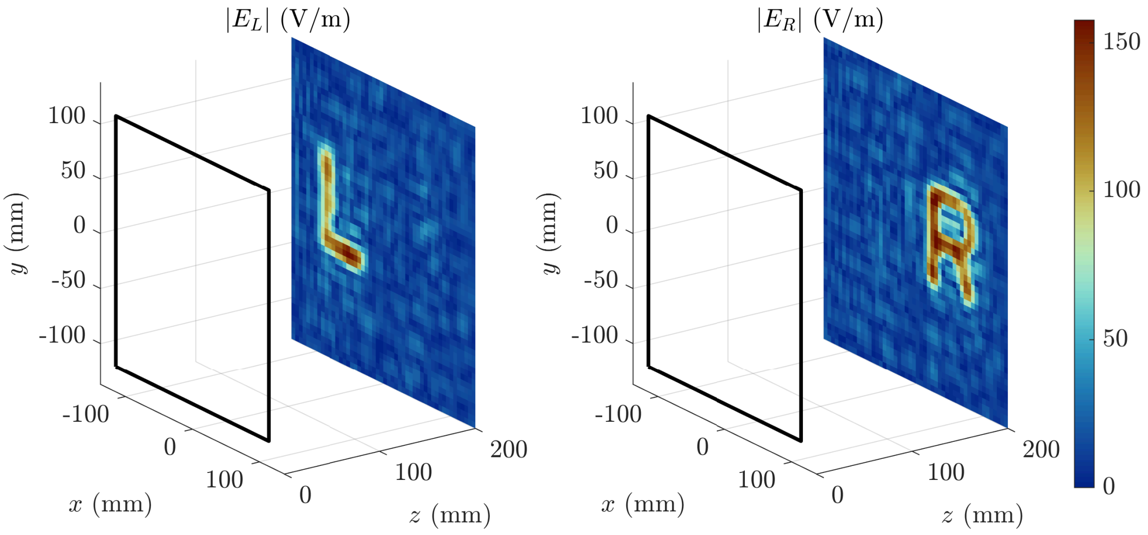

Figure S22: Electric field measurement at 200 mm from the metasurface whose contours are represented in black. (**Left**) Amplitude of the electric field in left circular polarization. (**Right**) Amplitude of the electric field in right circular polarization.

The field is initially measured with a linearly mono-polarized reference antenna. The latter is thus placed during two successive scans according to the horizontal and vertical polarizations (Fig. S23). In these representation basis, a projection of the letters formed by this hologram are visible in both cases but the associated depiction of the phase states makes it possible to reveal an almost constant phase according to the vertical polarization and a phase jump of $\pi/2$ between the two letters formed in horizontal polarization. The results of the measurements are displayed using a colormap developed to limit visual distortions and more easily interpretable by people with color vision deficiency [15].

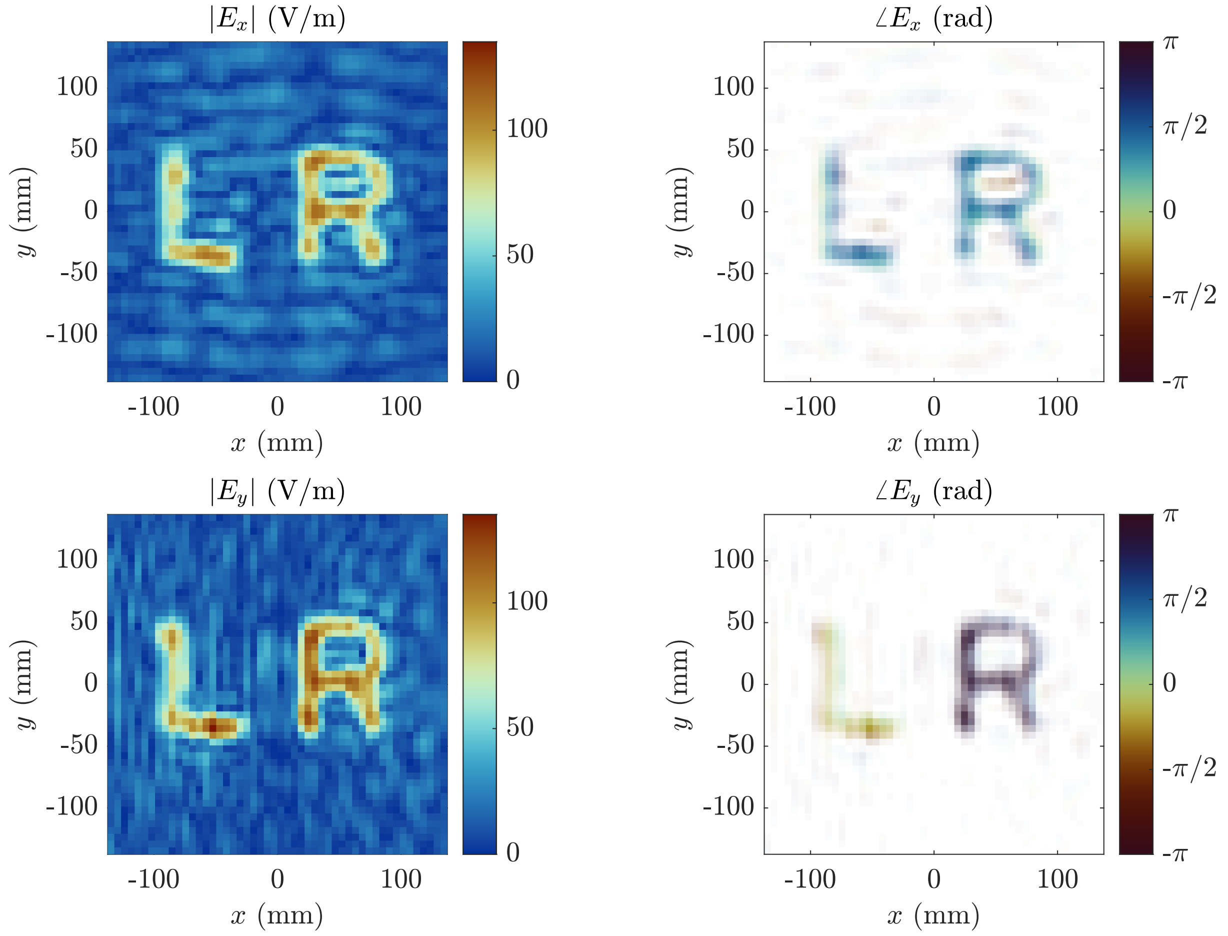

Figure S23: Field scans represented according to the vertical and horizontal polarizations as measured by the reference antenna used for this experimental demonstration. The two right sub-figures represent the phase distributions of the two scans, applying an opacity determined from the associated amplitudes.

Linear combinations of these two measurements can finally be computed to reveal electric field distributions according to left and right circular polarizations (Fig. S24). The representation of the phase information associated with the amplitudes presented in the paper reveals a quasi-constant phase state for both letters.

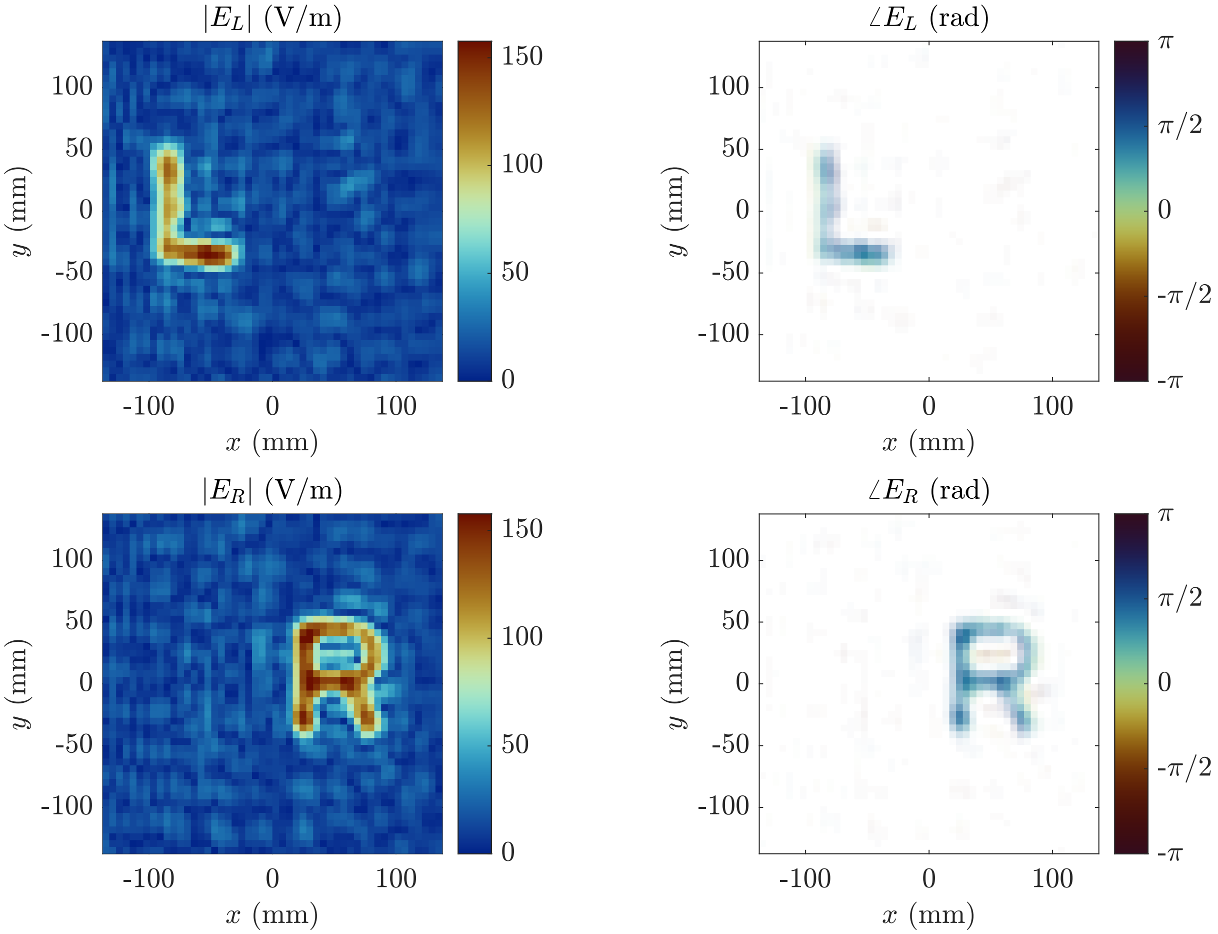

Figure S24: Electric field measurements shown in left and right-hand circular polarization respectively.

7 Positioning in relation to conventional techniques

Numerous works based on the exploitation of patterns arranged on uniform grids already exist in the literature. The most advanced proofs of concept exploit sophisticated mathematical formalisms, notably allowing the effect of impedance modulations on the surface wave to be taken into account in order to correct the objective tensors. These optimizations are obtained at the cost of the development of complex numerical techniques and the use of restrictions on metasurface geometries to facilitate domain decompositions, but have proved their effectiveness and define the state-of-the-art in metasurface antennas.

In this section, it is proposed to compare the performance of the morphogenetic generation technique with a conventional approach, consisting in exploiting patterns arranged on a uniform grid. In order to offer a comparison that is both fair and easy to comprehend, it has been necessary to simplify the constraints considered. It is therefore proposed to compare the performance obtained for two metasurfaces occupying an area of $200 \times 200\text{mm}^2$, radiating a single left-hand circularly polarized beam in a direction normal to the metasurfaces. The first-order approximations previously developed are once again used to facilitate these comparisons.

For this simplified comparison, the anisotropy of the objective tensors is kept constant. All the elliptical patterns generated for the conventional metasurface will therefore be of identical dimensions, and the morphogenetic parameters will be kept identical throughout the metasurface. In both cases, only rotations of the patterns will be allowed, either directly or via the diffusion tensors to ensure surface wave conversion.

It is therefore important to note that the performance obtained in each case can be greatly optimized. Under these simplified conditions, it will nevertheless be possible to easily illustrate the effect of geometric discontinuities linked to the exploitation of uniform grids of patterns, where the self-structuring capacity of morphogenetic patterns tends to provide a more homogeneous distribution of these spacings.

Firstly, it is proposed to compare the characteristics obtained for two patterns optimized to present comparable properties. The dimensions are chosen so as to ensure the synthesis of a reactance tensor whose average tends towards $X_{sw} = 270\Omega$ and whose anisotropy modulation tends to $a_X = 0.17$. The latter value is deliberately chosen to be lower than the maximum achievable by the morphogenetic method, in order to facilitate the synthesis of an equivalent characteristic by means of identical patterns arranged on a uniform grid. The first part of this study focuses on the effect of rotating the tensors to be synthesized under these simplified conditions. An illustration of the patterns generated in each case already reveals some significant differences (Fig. S25)

For a rotation series varying between $\varphi = 0°$ and $\varphi = 90°$, the tensors are determined

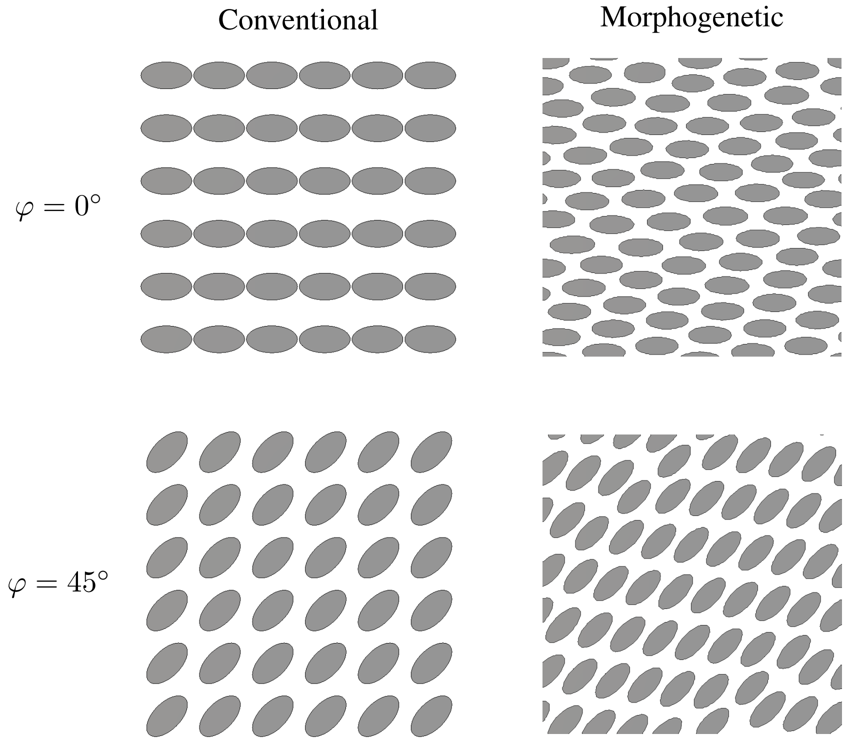

Figure S25: Patterns enabling the synthesis of reactance tensors of identical average and anisotropy for two rotations such as $\varphi = 0°$ and $\varphi = 45°$. The self-structuring capability of the morphogenetic technique results in a more regular distribution of elements.

following the procedure presented previously. The average values X_{sw} of the reactance tensors obtained for each rotation value, together with the a_X anisotropy, are presented in Fig. S26.

Under the effect of variations in distances between elements, the conventional approach undergoes a change in the synthesized characteristics. In line with the previous graphical justification, the properties obtained by the morphogenetic technique are more rotationally stable, guaranteeing better control of the properties obtained.

It is important to note that a homogenization technique was used to extract morphogenetic parameters. As the synthesis was carried out in a domain with periodic boundary conditions, initial results tended to indicate a strong dependence of pattern rotation on extracted properties. Due to the repulsion of elements on shared boundaries, the compactness of the resulting arrangements is influenced by the orientation of the eigenaxes. To limit these boundary condition effects and extract only the features associated with the patterns, the characterization space was increased to $20 \times 20\text{mm}^2$. Furthermore, the

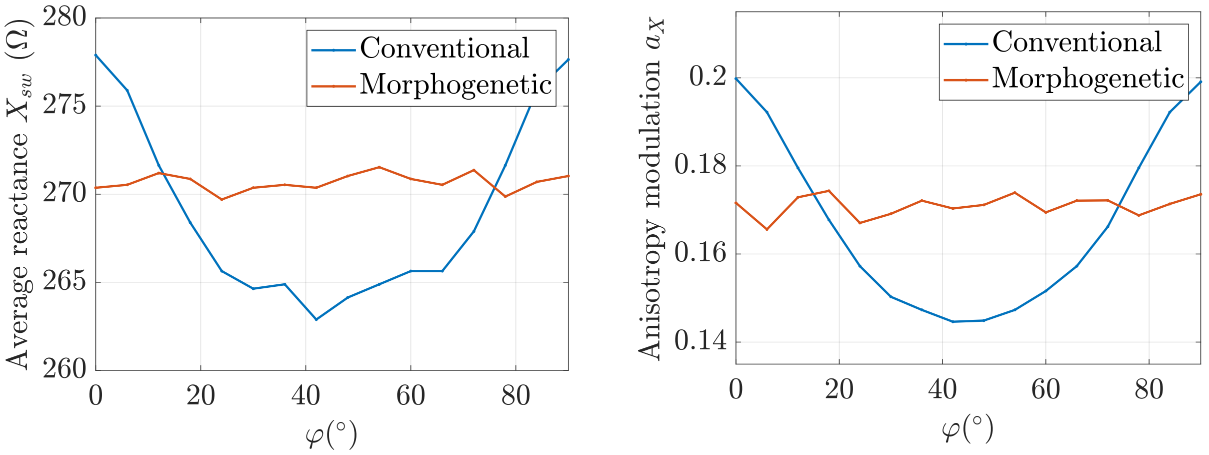

Figure S26: Effect of geometric discontinuities on the variation in the characteristics of the reactance tensors generated.

results presented are obtained by averaging 10 random initializations. Finally, these results are averaged for two orientations of the Floquet ports (following a 45° rotation), considering that results from a tensor diagonalization are assumed to be invariant with the rotation of the eigenaxes chosen for excitation. This property was verified for elliptical patterns arranged on a uniform grid, where rotation of the eigenaxes of the Floquet ports had no effect on the properties extracted after tensor diagonalization.

Two metasurfaces are synthesized from these patterns according to the specifications previously outlined (Fig. S27). These representations highlight the smaller dimensions of the patterns generated by reaction-diffusion compared with those obtained for uniform paving, recalling that the design was carried out to obtain the same average reactance and anisotropy characteristics. This difference in dimensions is justified by the stronger interaction of the Turing patterns with neighboring elements. It should be noted, however, that the smallest spacings between these patterns remain generally greater than those obtained for the conventional approach when the ellipses are aligned horizontally, thus facilitating the fabrication of morphogenetic metasurfaces.

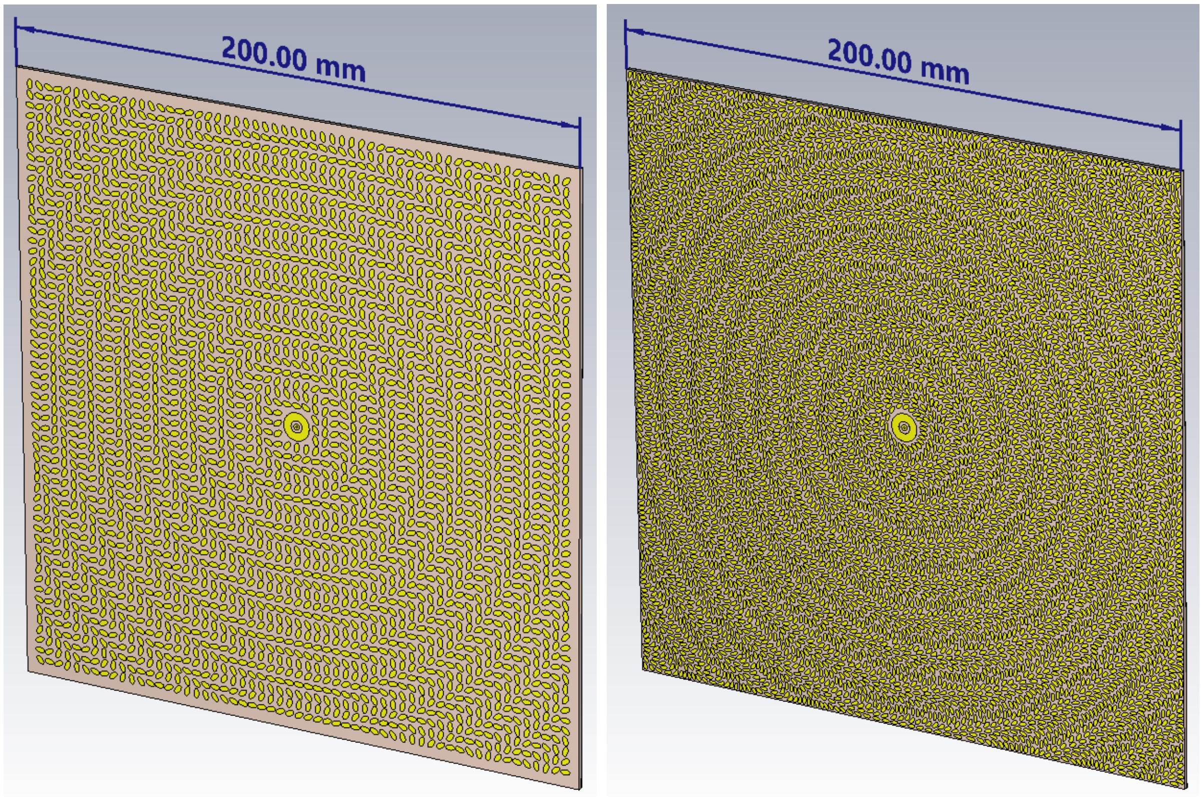

Figure S27: Conventional (left) and morphognetic (right) metasurfaces designed according to the same specifications to radiate a beam normal to the antennas, circularly polarized to the left. The patterns are chosen in each case to provide the closest possible average characteristics.

The computation of radiation highlights the effect of better control of the synthesized reactance tensors (Fig. S28). With these two basic designs, radiation is well achieved in the desired direction and polarization state. However, the morphogenetic method guarantees better control of polarization, both in terms of the level of secondary lobes in left circular polarization and overall control of cross-polarization.

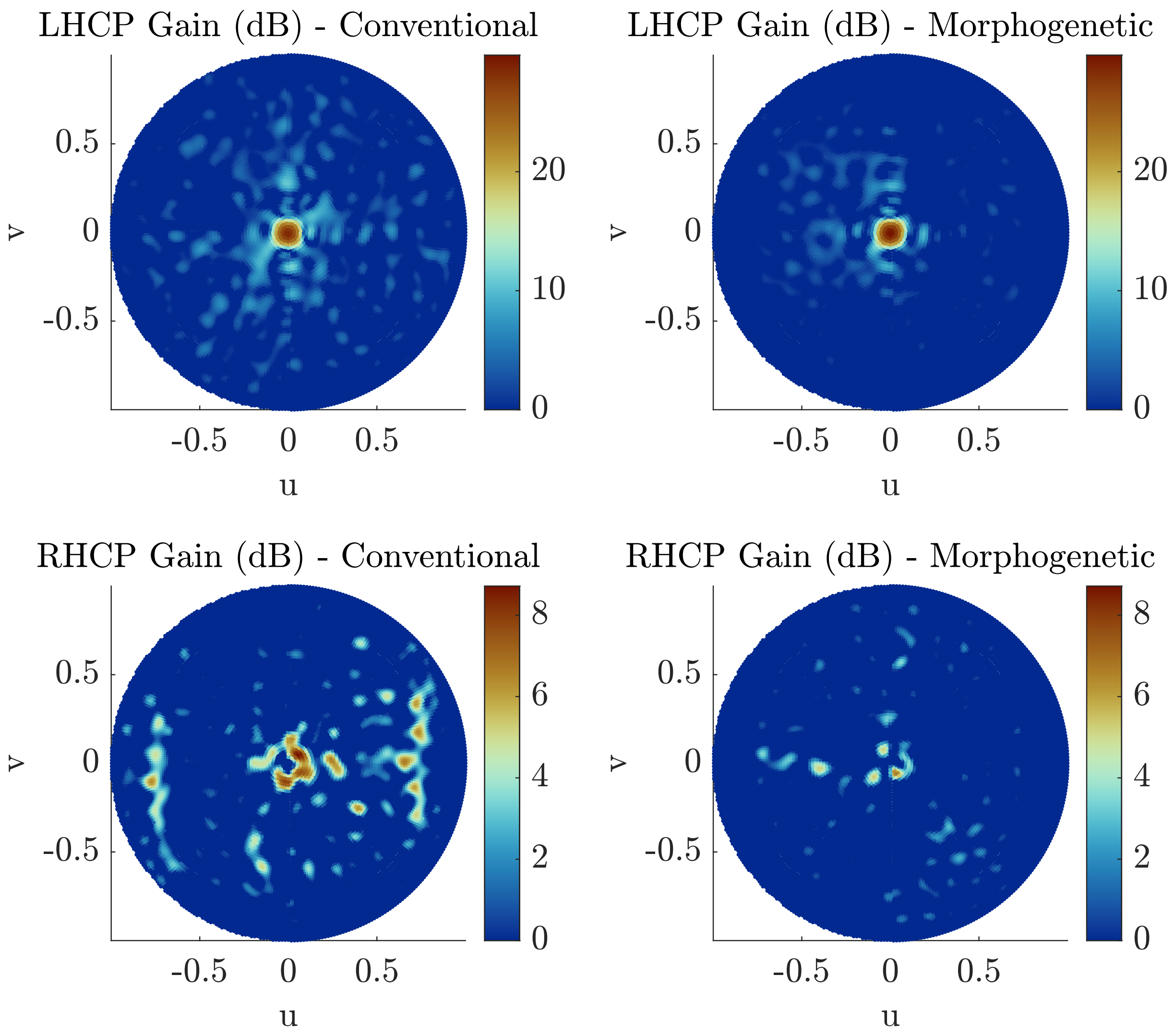

Figure S28: Improved anisotropy control through the morphogenetic approach enables more selective management of the radiated polarization state. The color bar maximum is adjusted according to the maximum gain obtained for the pair of metasurfaces, ensuring that the colors correspond to identical gain levels. Data are displayed for the frequencies corresponding to the maximum gains for each case, i.e. 19.5 GHz for the conventional metasurface and 20 GHz for the morphogenetic metasurface.

With a more homogeneous distribution of the generated patterns, a slight increase in on-axis gain is finally obtained with this demonstration (29.8 dB of maximum gain for the morphogenetic metasurface versus 26.8 dB for the conventional case), as well as a widening of the operating band (1.3 GHz at -3 dB from the maximum for the morphogenetic case versus 1.15 GHz for the conventional one) in connection a stronger effet of periodicity breaks in the conventional approach (Fig. S29).

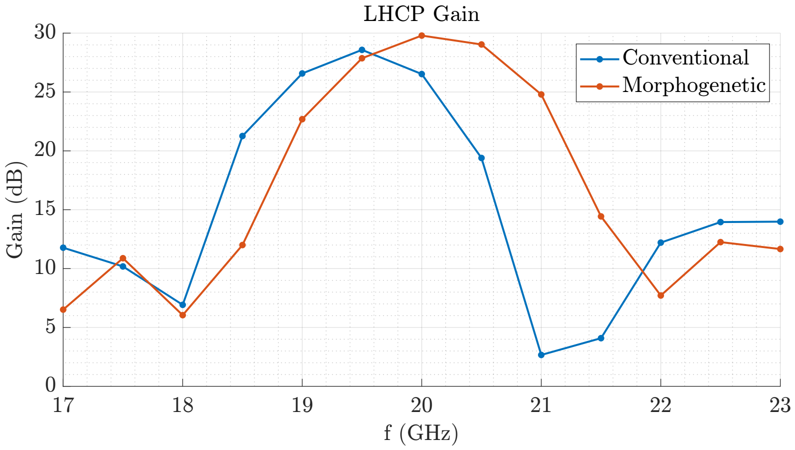

Figure S29: Under the simple conditions proposed, the morphogenetic approach enables an increase in gain, a better frequency control, and a widening of the bandwidth compared with the conventional approach.

Considering the simplicity of this demonstration, both approaches can obviously be greatly optimized, but a comparison carried out under simple, controlled conditions nevertheless highlights the contributions of the proposed technique. The multiplexing of radiation targets, the optimization of aperture efficiency and the synthesis of holograms tend to impose much stronger constraints on the spatial modulation of anisotropy, implying even greater constraints on the arrangement of the resulting meta-atoms (Fig. S30). Further investigations will enable us to quantify in finer detail the advantages offered by the proposed technique over conventional approaches in these more advanced cases.

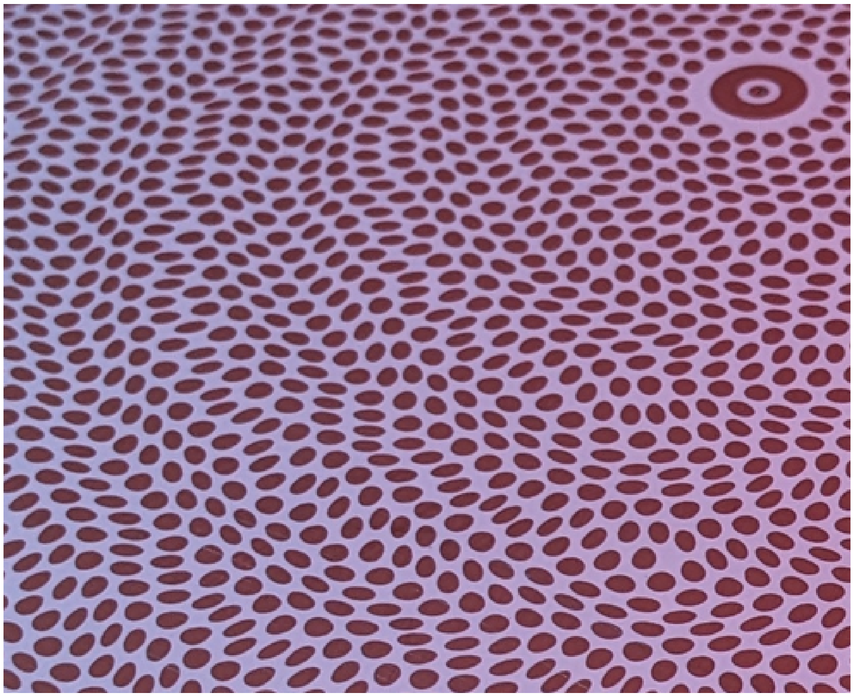

Figure S30: Zoom in on a distribution of meta-atoms, highlighting the self-structuring capacity of the proposed morphogenetic technique.

Supplementary References

[1] E. Martini, M. Mencagli Jr, S. Maci, *Philosophical Transactions of the Royal Society A: Mathematical, Physical and Engineering Sciences* **373**, 20140355 (2015).

[2] T. Brown, C. Narendra, Y. Vahabzadeh, C. Caloz, P. Mojabi, *IEEE Transactions on Antennas and Propagation* **68**, 1812 (2019).

[3] B. H. Fong, J. S. Colburn, J. J. Ottusch, J. L. Visher, D. F. Sievenpiper, *IEEE Transactions on Antennas and Propagation* **58**, 3212 (2010).

[4] G. Minatti, *et al.*, *IEEE Transactions on Antennas and Propagation* **63**, 1288 (2014).

[5] J. Cavillot, M. Bodehou, C. Craeye, *IEEE Transactions on Antennas and Propagation* (2022).

[6] N. A. Rubin, A. Zaidi, A. H. Dorrah, Z. Shi, F. Capasso, *Science Advances* **7**, eabg7488 (2021).

[7] A. M. Turing, *Bulletin of mathematical biology* **52**, 153 (1990).

[8] P. Gray, S. Scott, *Chemical Engineering Science* **38**, 29 (1983).

[9] P. Scarabotti, T. Govezensky, P. Bolcatto, R. A. Barrio, *Scientific Reports* **10**, 1 (2020).

[10] A. Witkin, M. Kass, *Proceedings of the 18th annual conference on Computer graphics and interactive techniques* (1991), pp. 299–308.

[11] T. McGraw, *Computers & Graphics* **32**, 82 (2008).

[12] M.-T. Chi, W.-C. Liu, S.-H. Hsu, *The Visual Computer* **32**, 1549 (2016).

[13] A. M. Patel, A. Grbic, *IEEE transactions on antennas and propagation* **59**, 2087 (2011).

[14] M. Faenzi, *et al.*, *Scientific reports* **9**, 1 (2019).

[15] F. Crameri, *Zenodo* **10** (2018).
