# Supplementary material for: Morphogenetic metasurfaces: unlocking the potential of Turing patterns
Source: Nat Commun. 2023 Oct 6;14:6249. doi: 10.1038/s41467-023-41775-9 (PMC10558543; doi:10.1038/s41467-023-41775-9)
Supplement: Supplementary file 3 — Description of Additional Supplementary Files [file 41467_2023_41775_MOESM3_ESM.pdf]

Description of Additional Supplementary Files

Supplementary Movie 1: Single polarized morphogenetic metasurface. Procedural generation of a singlepolarized morphogenetic metasurface.

Supplementary Movie 2: Dual polarized morphogenetic metasurface. Procedural generation of a dual circularly polarized in two different directions.

Supplementary Movie 3: Hologram measurement. Representation of the electric field measured at 20.2 GHz, revealing the direction of rotation of the polarization of distributions forming the letters L and R.
